# Guard cell and whole plant expression of *At*TOR improves performance under drought and enhances water use efficiency

**DOI:** 10.1016/j.jbc.2025.110220

**Published:** 2025-05-13

**Authors:** Li Liu, Peng Gao, Huajin Sheng, Achala Bakshi, David Schneider, Daoquan Xiang, Vivijan Babic, Maozhi Ren, Connor Burbridge, Hanh Nguyen, Sheng Wang, Alma Armenta-Medina, Javier Mora-Macias, Andrew Sharpe, Curtis Pozniak, Jurandir Magalhaes, Raju Datla, Leon Kochian

**Affiliations:** 1 Global Institute for Food Security, University of Saskatchewan, Saskatoon, Saskatchewan, Canada; 2 Aquatic and Crop Resource Development, National Research Council Canada, Saskatoon, Saskatchewan, Canada; 3 Institute of Urban Agriculture, Chinese Academy of Agricultural Sciences, Chengdu, China; 4 Centro Nacional de Recursos Geneticos, Instituto Nacional de Investigaciones Forestales, Agricolas y Pecuarias, Tepatitlan de Morelos, Jalisco, Mexico; 5 Crop Development Centre, University of Saskatchewan, Saskatoon, Saskatchewan, Canada; 6 Embrapa Maize and Sorghum, Sete Lagoas, Minas Gerais, Brazil

**Keywords:** arabidopsis, drought resistance, ectopic expression, guard cell, leaf conductance, photosynthesis, *TOR*, transpiration, water use efficiency

## Abstract

Water use efficiency is an important target for breeding of improved drought resistance. Minimizing leaf transpirational water loss plays a key role in drought resistance. But this reduces CO_2_ levels in leaves, which often reduces photosynthetic efficiency and yield. Signaling pathways play important roles in stress responses, and identifying the molecular, biochemical, and physiological determinants underlying drought signaling may offer new drought mitigating strategies. To explore these possibilities, and because of the importance of stomata in drought response and photosynthesis, we employed guard cell (GC)-targeted and constitutive overexpression of the Target of Rapamycin (TOR) kinase, a master regulator of signaling networks, in transgenic Arabidopsis. To investigate the impact of these *AtTOR* transgenes in drought, we conducted physiological and molecular investigations into drought responses, including leaf water loss, photosynthetic CO_2_ assimilation, stomatal H_2_O/CO_2_ conductance, leaf chlorophyll content, and global gene expression in response to drought in wild-type and *AtTOR*-expressing Arabidopsis. Links between both guard cell-localized and whole plant *AtTOR* overexpression were identified, revealing TOR is involved in conservation of water and sustained photosynthetic performance, along with identification of genes associated with drought response in WT versus *AtTOR*-expressing transgenic lines. These findings suggest that targeted guard cell *AtTOR* expression should help achieve a balance between plant water conservation during drought, and maintaining plant performance, by minimizing reductions in photosynthesis. Manipulation of guard cell *AtTOR* expression could be an effective avenue for developing crops with enhanced drought resistance and increased yield under drought stress, resulting in enhanced water use efficiency.

Water-use efficiency (WUE), broadly defined as the water used by the plant for per unit of biomass or grain yield produced from that unit of water, has been considered a key target for crop improvement (1). The essential plant processes of carbon dioxide (CO_2_) assimilation for photosynthetic reactions and water loss through evapotranspiration directly determine WUE, and this is influenced in part by the precise regulation of leaf stomatal conductance (2, 3). Drought resistance in crops, which can be considered the adaptive capability of some crop plant genotypes to survive and maintain reasonable yields under water limiting conditions, is often associated with lower transpiration through reduced stomatal opening, which reduces water loss. There is generally a cost associated with this type of stomatal behavior that underlies WUE, as it also leads to reduced stomatal flux of CO_2_ into leaf tissues and cells, leading to lower leaf CO_2_ concentrations. This, in turn can lead to reduced photosynthetic carbon assimilation and photosynthetic performance, resulting in yield reductions (4). As WUE is often equated in a relatively simplistic manner with crop drought resistance, equipping crop plants with improved WUE is often considered an important target for crop protection and improvement (5), which must also overcome the possible negative impact on photosynthesis. This is a research area increasing in importance, given the impact in recent years of climate change, resulting in more frequent and severe episodes of drought. To address this emerging agricultural problem, there has been an increased interest in both the identification and characterization of important molecular and physiological processes involved in WUE and improved performance under drought in experimental model plants, especially *Arabidopsis thaliana*, and subsequently using our enhanced understanding of WUE obtained from Arabidopsis to explore if similar mechanisms can be manipulated in important crop plants, to increase crop yields in response to drought (6, 7, 8, 9, 10). Previously, transgenic approaches were used in wheat where overexpression of an abscisic acid (ABA) receptor improved WUE and drought resistance, WUE was improved during water deficit due to reduced stomatal aperture size resulting in lower transpiration rates, and also a surprising increase was quantified in photosynthetic activity, for which the underpinning mechanisms are not known (8). In transgenic Arabidopsis, research on WUE was conducted *via* the over-expression of an epidermal patterning factor (EPF), which reduced stomatal density and stomatal conductance, potentially improving WUE (11). Thus, the development of other gene-based tools that could create an effective balance between photosynthetic performance and regulation of transpirational water loss will provide new strategic opportunities for equipping and adapting crops to environmental challenges associated with drought stress.

Target of Rapamycin (TOR) is a member of the phosphatidylinositol 3-kinase-related protein kinase superfamily, encoding a serine-threonine protein kinase that is evolutionarily conserved in all eukaryotes, where the TOR protein has five conserved protein domains (12, 13, 14). In plants, *TOR* has been shown to be involved in diverse and important processes, including growth regulation, development, ribosome biogenesis, protein synthesis, and metabolism. TOR thereby plays important roles at every stage of plant life, from embryogenesis to meristem activation, vegetative and reproductive growth, senescence, and life span determination (15, 16, 17, 18, 19), where TOR orchestrates the linking of nutrient and energy status to cell growth and metabolic homeostasis. Recent studies in Arabidopsis, rice, and cotton have also uncovered roles for TOR in response to abiotic stresses and regulation of photosynthesis (20, 21, 22, 23). These studies primarily focused on expression analyses of selected genes along with the use of plant-wide overexpression of *TOR via* the constitutive *CaMV 35S* promoter, which understandably makes it difficult to untangle and define the roles of *TOR* in balancing abiotic stresses and cell growth responses, because ectopic overexpression of *TOR* in some tissues where it normally is not highly expressed might alter this balance.

To address this issue, we have used a more targeted approach to regulate ectopic expression of *TOR* by generating Arabidopsis transgenic lines where *AtTOR* expression was targeted to guard cells using a guard cell-specific promoter. Guard cells were chosen as the *AtTOR* expression target as our previous research (20) suggested that TOR was involved in plant water relations, particularly during drought stress. Since guard cells are well known for their important functional roles in adapting to drought stress, this was a logical place to start regarding targeted plant *TOR* expression. As a comparison, we also employed constitutively over-expressed *AtTOR* driven by the CaMV 35S promoter, which is known to express genes in different leaf tissues, including guard cells, where its expression overlaps with the guard cell specific promoter driving *AtTOR* expression. These two different Arabidopsis transgenic lines and the control WT lines, were then characterized for plant development, as well as physiological and molecular aspects of leaf transpiration rates, CO_2_ assimilation, stomatal conductance, canopy temperature, global gene expression analysis and WUE, under *normal vs*. drought-challenged conditions. These studies revealed that ectopic whole plant *AtTOR* expression (designated as OE) as well as increased *AtTOR* expression localized to guard cells, both resulted in improved plant performance and recovery from drought, *via* reduced transpiration and increased photosynthetic efficiency. The research findings presented here show that *AtTOR* expression specifically only in guard cells shows promise as an effective strategy for improving WUE *via* transgenic and recently emerging gene editing approaches, in a way that should allow future studies that identify the likely quite complex mechanisms underlying *AtTOR*-mediated enhancement of drought resistance.

## Results

### Expressing *AtTOR* specifically in guard cells and throughout the plant

In response to environmental stress, plants activate complex signaling programs to mount a range of protective stress responses. In this study, we investigated the possible roles of one of presumably multiple signaling pathways mediated by the Target of Rapamycin (TOR) kinase that may modulate responses to drought stress. To elucidate the possible physiological mechanism(s) by which TOR is involved in improved plant performance in response to water deficiency stress, we conducted physiological studies on Arabidopsis WT and transgenic lines where the full-length *AtTOR* was under the control of one of several promoters we had previously isolated and characterized (24). For the studies described here, independent transgenic plants expressing the full-length *AtTOR* driven by 35S or guard cell-specific promoters were generated (as described in detail in the Materials and Methods; and Fig. S1).

**Figure 1:**
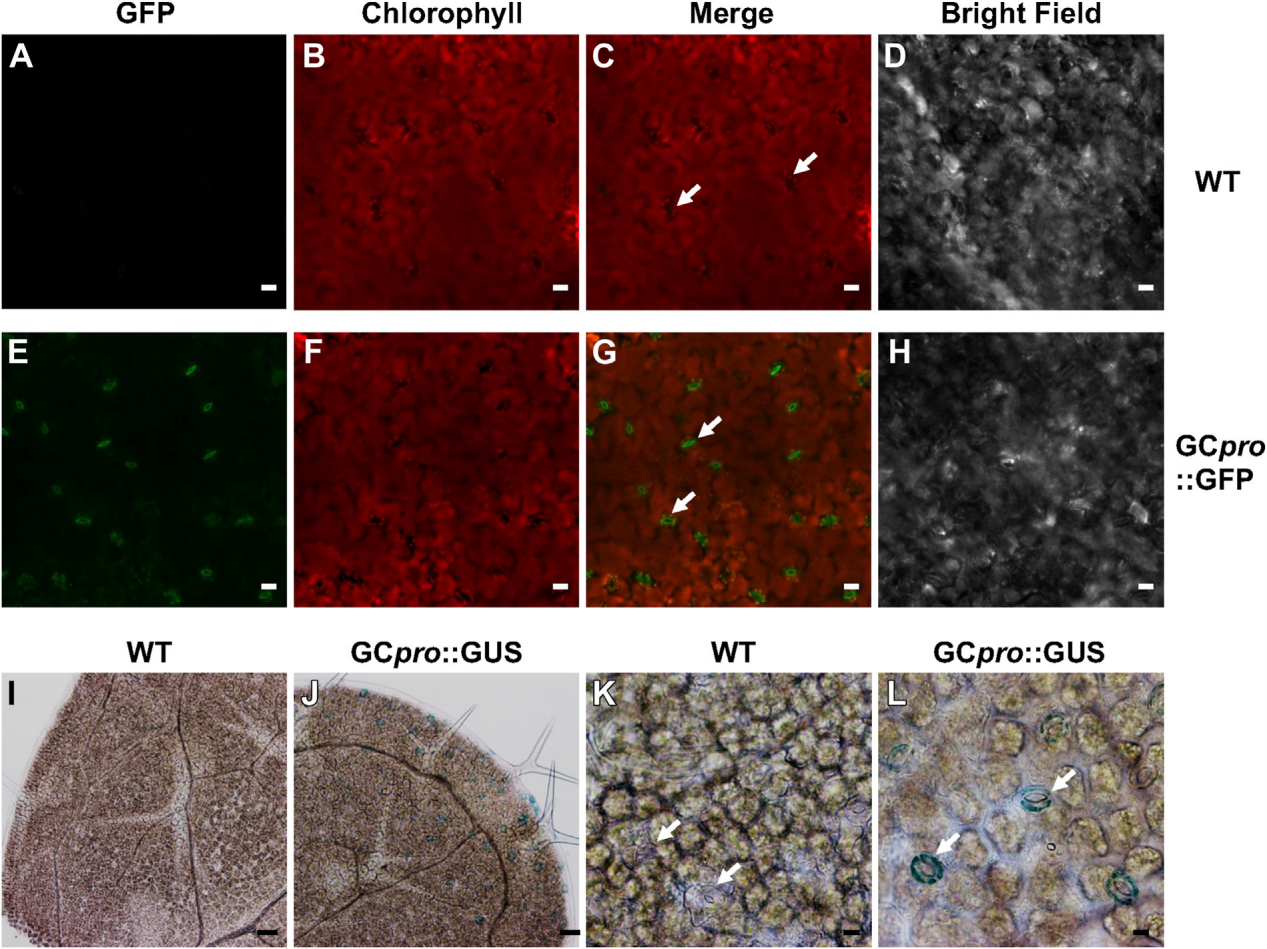
**Guardcell-specific promoter drives ectopic expression of TOR in Arabidopsis.***A*–*H*, GC promoter fused to green fluorescence protein (GFP) was used to evaluate the localization of the construct in Arabidopsis leaves for plants grown in growth medium under normal conditions. *White bar* = 10 μm. *I*–*L*, GC promoter fused to the *β-glucuronidase* (*GUS*) reporter gene showed that using a second reporter gene yielded the same guard cell-specific *TOR* expression in Arabidopsis leaves. *White* arrows indicate representative stomata in both wildtype (WT) and transgenic plants. *Black bar* for *I* and *J* = 50um, and *black bar* for *K* and *L* = 10um.

Guard cells are involved in important physiological processes associated with leaf transpiration and CO_2_ uptake, which play vital functions in photosynthesis as well as water flux through the plant under normal and drought-challenged conditions. The guard cell-specific promoter used in this study was isolated and characterized from tobacco (24), and is located upstream of a RAP2.4-like ethylene-responsive transcription factor (LOC107774257, in *Ntab-TN90*_scaffold3543), and as shown in Figure 1, it drives guard cell-specific expression. We first produced transgenic lines with the tobacco GC promoter fused to the green fluorescence protein (GFP) reporter and evaluated the localization of GFP expression in plants grown under normal, well-watered conditions. In 5 independent lines, GFP-specific signals were detected only in guard cells (Fig. 1, *A*–*H*). A second set of transgenic lines containing the GC promoter fused to β-glucuronidase (GUS) were also constructed and evaluated for further confirmation of this promoter’s guard cell-specific expression. Strong and specific GUS reporter activity was visible only in guard cells of stomata in all lines grown under normal, well-watered conditions (n = 2) (Fig. 1, *I*–*L*). No detectable GUS or GFP signal was observed in other leaf cell types or tissues, confirming that the promoter drives reporter gene expression specifically in guard cells. Given our preliminary findings in Figure S2 showing that *AtTOR* driven by either the 35S or GC-specific promoter exhibited improved Arabidopsis drought resistance, and the confirmation that GC promoter-driven regulation of *AtTOR* expression localizes specifically to guard cells, strongly suggests that *AtTOR* expression in the guard cells alone is sufficient to induce the improved performance in response to the drought stress conditions used in this study. As the CaMV35S promoter also drives expression in guard cells as well as in most other plant cells, similar drought responses are expected with both promoter constructs. These observations are consistent with the well-documented key functions of guard cells comprising the stomatal complex in regulating transpirational water flux out of leaves and CO_2_ flux into leaves (11).

### *TOR* transgenes improve Arabidopsis performance in response to drought

Transgenic plants expressing either *35S*::*AtTOR* or *GC*::*AtTOR* were generated using the floral dip method commonly used in Arabidopsis (25), and the control was the same wildtype (WT) *Col-0* ecotype that was transformed. The transgenic and WT *Col-0* ecotypes were evaluated in experiments for performance in response to drought. This involved growing the WT and transgenic plants under well-watered long-day conditions for up to 27 days before applying the drought challenge, which involved the withholding of water for 11 days (Fig. S2). Among all of the transgenic plants in this experiment, 67% of the *35S*::*AtTOR* and 100% of the *GC*::*AtTOR* lines survived the drought-challenged stress period, while all the WT plants were killed by the same drought stress conditions, showing that drought resistance was significantly increased by overexpression of *AtTOR* driven by either the *35S* or *GC* promoters.

**Figure 2:**
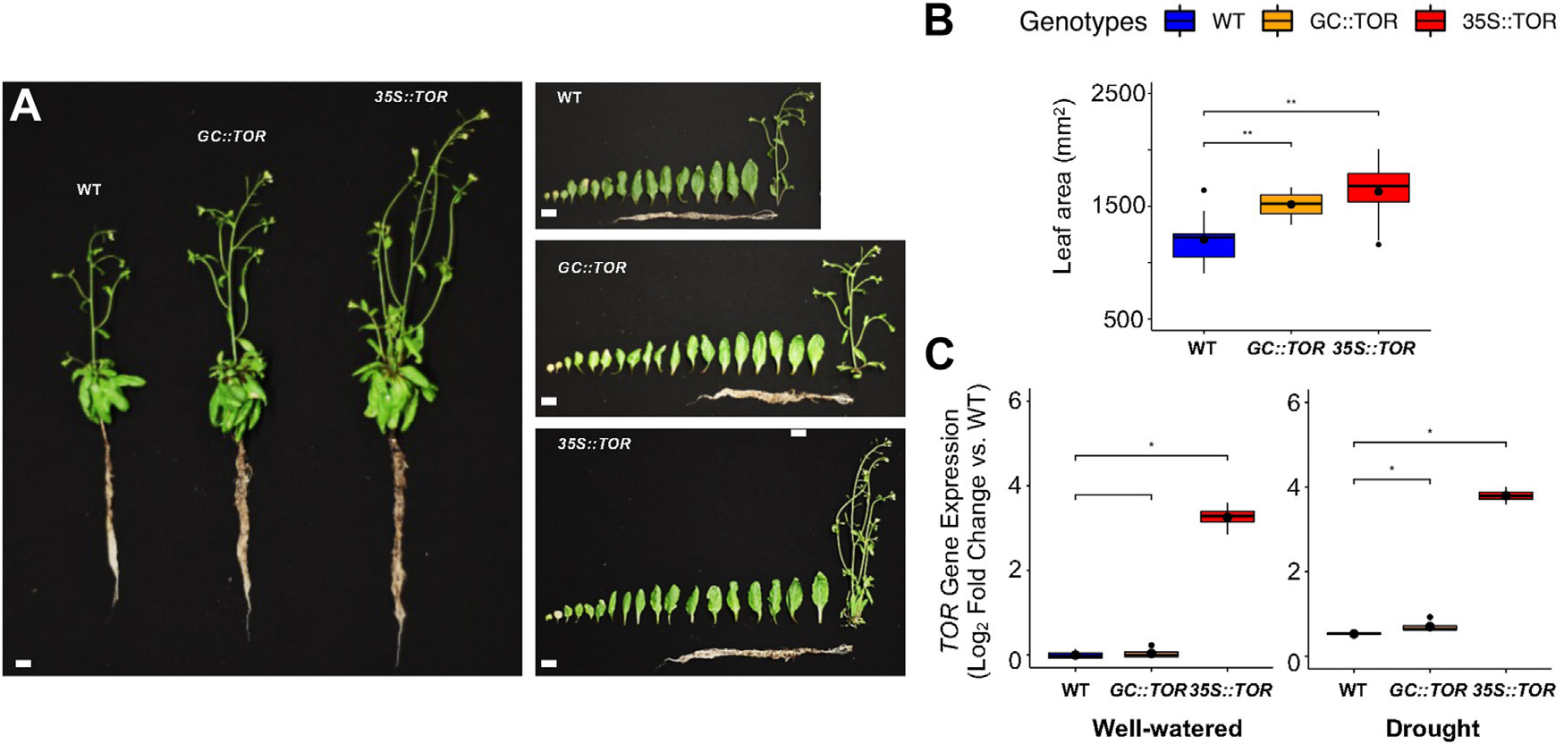
**Phenotypic analysis of Arabidopsis WT and transgenic lines expressing full-length *AtTOR* driven by *35S* and *GC* promotors.***A*, images of the whole plant, rosette leaves, inflorescences and roots of WT, *GC::AtTOR* and *35S::AtTOR* lines at 50 DAG under long-day (16 h light; to induce flowering) and well-watered conditions. Transgenic plants exhibited earlier flowering and faster leaf development, as well as a larger root system compared with WT plants, when all were grown under well-watered conditions. Scale bar = 1.6 cm. *B*, total leaf area was measured for WT, *GC::AtTOR* and *35S::AtTOR* lines grown under short-day (8 h light; to study vegetative growth) and well-watered conditions and the leaf area data is presented as box plots. The larger *black circle* within the box of each box plot represents the mean value for leaf area, and the smaller *black circles* beyond the whiskers represent outliers. Plants are 35 DAG in age. The leaf area of the transgenic lines, *GC::AtTOR* and *35S::AtTOR*, were significantly larger than WT leaf area. *C*, *TOR* gene expression detected by ddPCR, with the actin 2 gene used as a reference. Relative expression level (*TOR/Actin 2*) was calculated for each genotype under well-watered and drought conditions, respectively. Relative expression data is presented as box plots. The *black circle* within the box of each box plot represents the mean value for leaf area and the *black circle* beyond the whiskers represent outliers. Four biological replicates were used for each genotype. In *C*, the low *TOR* gene expression in the *GC::AtTOR* lines compared to *35S::AtTOR* expression is due to the very small number of guard cells expressing *AtTOR* compared to TOR expression in all of the leaf cells. Analysis of variance of the data was conducted using the student’s *t* test. ∗, 0.01 < *p* < 0.05; ∗∗, 0.001 < *p* < 0.01.

Plant growth and development were then assessed in more detail for each of the *35S*::*AtTOR* and *GC*::*AtTOR* lines at 50 days after germination (DAG), with the plants grown under well-watered conditions for those first 50 days. Compared to WT plants, well-watered transgenic plants exhibited early flowering, faster leaf development, and the development of a larger root system (Fig 2*A*). Also, the ectopically expressed lines had significantly greater total leaf area compared to WT plants (Fig. 2*B*). When many plants were measured, there was not a statistically significant difference in total leaf area between the *35S*::*AtTOR* and *GC*::*AtTOR* lines (Fig. 2*B*).

To detect gene transcript abundance changes of *AtTOR* in transgenic lines, we performed Droplet Digital PCR (ddPCR) assays with an *AtTOR-specific* probe (Table S1), using rosette leaf RNA samples at 62 DAG under short-day conditions. Transcript copy per droplets (CPDs) of both *AtTOR* and the housekeeping reference gene, *Actin 2*, were calculated for WT and *AtTOR* transgenic lines, respectively. Compared to WT, *35S*::*AtTOR* lines exhibited significantly increased *AtTOR* expression under both well-watered and drought conditions (Fig. 2*C*). However, *GC*::*AtTOR* lines showed similar *AtTOR* expression levels as was seen in WT leaves (Fig. 2*C*), which was expected, as guard cells make up only a very small percentage of the total cells in a leaf, so the presumed higher ectopic expression in transgenic guard cells would be diluted by the typically low *AtTOR* expression in the rest of the leaf cells.

**Figure 3:**
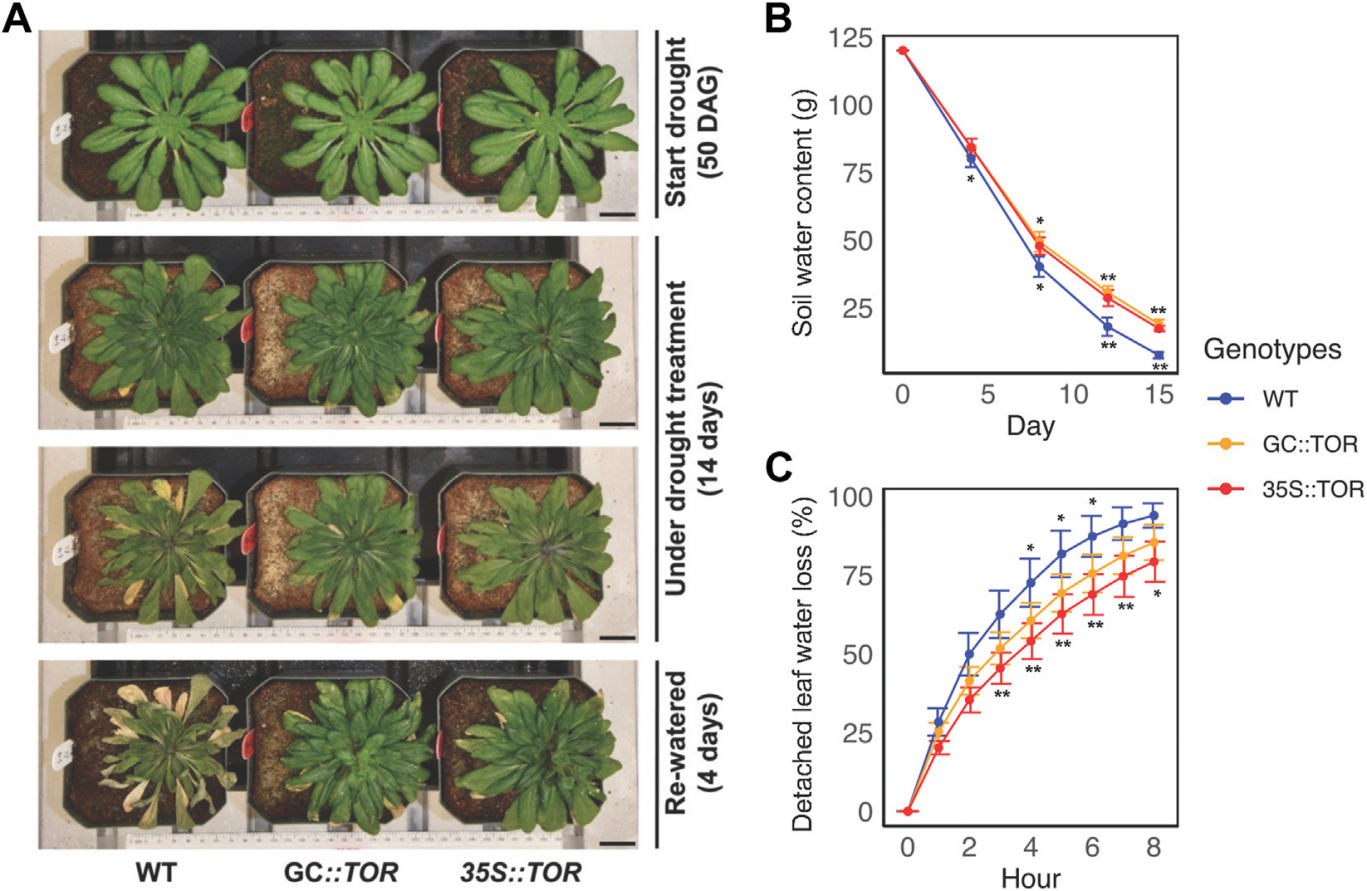
**Full-length *AtTOR* transgenic lines exhibited improved drought resistance during vegetative growth.***A*, drought treatment assay under short-day conditions. WT, *GC::AtTOR* and *35S::AtTOR* plants were grown under well-watered conditions for 50 DAG and then water was withheld for 14 days, under short-day growth conditions (8 h light). At the end of the 14-days drought period, watering was resumed for 4 days. From *top* to *bottom*, the representative images show: plants 50 DAG just before the start of the drought treatment (first row), plants after 7 days of drought treatment (second row), plants after 14 days of drought treatment (third row), and plants 4 days after watering was resumed (last row). *GC::AtTOR* and *35S::AtTOR* exhibited greater drought resistance than the WT plants. After re-watering, the *AtTOR* transgenic lines quickly recovered and rapidly began reproductive growth which enabled the plants to flower and produce significant seed, while the WT plants exhibited no recovery during re-watering and appear to be dead data not shown). Scale bar = 2.5 cm. *B*, time course of transpirational water loss from intact plants grown in potting mix over 15 days of drought (water withheld starting at day 0 shown on graph). When the drought treatment was initiated, the soil water content of each pot was set equally to 120g (60% of full soil water capacity) in WT, *35S::AtTOR* and *GC::AtTOR* lines. X-axis, days after drought treatment; Y-axis, measurement of the values of soil water content in grams of weight per pot. The total amount of water transpired at each drought time point (days 4, 8, 12 and 15) was determined by weighing the pot with its potting mix and plant at each time point, and the weight for each time point was subtracted from the day 0 time point, when the well-watered plant was at 60% of full soil water capacity. *C*, assay of the time course of detached leave’s transpirational water loss during the 8 h after leaves were excised. Leaves came from individual plants for each of the three genotypes of plants that were watered for 50 days and then leaves were excised from the plants and placed in small preweighed open plastic containers (two leaves per container, leaf abaxial side up). Containers were weighed every 60 min for 8 h. The experiments were repeated three times. At least five independent plants were used as 5 biological replicates for each genotype. Percentage of leaf water loss was calculated by 1 - (the leaf weight measured at each time point/the weight of the same leaf weighed just after it was excised). The excised leaves of WT plants lost more water than the leaves from the *AtTOR* transgenic lines, and this difference was statistically significant for measurements made hourly from hours 3 to 7 of the 8-h measurement period. X-axis, hours after leaves were excised; Y-axis, percentage of leaf water loss. For the data in Figure 3, *B* and *C*, standard derivations were calculated for each time point between WT and both type of *AtTOR* transgenic lines. Analysis of variance of the data was conducted using the Student’s *t* test. ∗, 0.01 < *p* < 0.05, ∗∗, 0.001 < *p* < 0.01 (top ∗: *GC::AtTOR* lines vs. WT, bottom ∗: *35S::AtTOR* lines vs. WT).

As short-day growth conditions allow longer periods of vegetative plant growth compared to the experiments described above for long-day plants (26), the drought experiments described here were performed with plants grown under short days. Drought was imposed by withholding water for 14 days after the plants had been grown for 50 days under well-watered conditions. At the end of the 14-day drought period, water was again provided for 4 days. As shown in Figure 3*A*, in response to drought, the representative *35S*::*AtTOR* and *GC*::*AtTOR* transgenic lines exhibited fewer senescent and necrotic leaves and more advanced plant development compared to WT plants. This was particularly evident following the reintroduction of water, where both types of *AtTOR-expressing* transgenic lines rapidly recovered and then subsequently flowered and produced seeds (data not shown). By comparison, the WT plants failed to recover upon the resumption of watering, suggesting that in addition to the observed leaf senescence and damage, WT plants were dramatically affected by drought, likely due to drought-induced damage to shoot meristems, inhibiting inflorescence and reproductive development.

To show the dynamic response of WT and transgenic plants to drought and then recovery after rewatering, we generated a time-lapse video to record the entire response through the 14-day drought treatment that was imposed starting at 50 DAG and then through the plant recovery period when the plants were rewatered (Movie S1). In this movie, besides the drought response, diurnal leaf responses to the light photoperiod can also be observed. Differential responses for inflorescence development and elongation in response to drought stress between WT and transgenic lines were also captured in the video, clearly showing the significant inhibition of reproductive growth by drought stress in the WT plants and the dramatic recovery of the transgenic plants to rewatering (Movie S1).

The overexpression of *AtTOR* using the *35S* promoter not only increased drought resistance and promoted rapid recovery from drought when re-watered, but it is clear that *AtTOR* expression also had a positive effect on growth and development under well-watered conditions, as seen in Figure 2, *A*–*C*. To avoid confusion between the 2 promoters used to drive TOR expression, we will use the term *AtTOR* ectopic expression to mean both *35S::*TOR and *GC::TOR* expressing lines. Taken together, these data suggest that *AtTOR* ectopic expression plays multiple important roles during plant growth and development, in addition to enhancing drought stress resistance, which is shared in and overlaps between these two transgenic lines.

### *AtTOR* enhances drought resistance by reducing transpirational water loss

Enhanced drought survival can be associated with a reduction in water loss, enabling the plant to conserve water during the drought period, and then recovery begins with a resumption of growth when water is resupplied. To evaluate the impact of *AtTOR* expression on transpirational water loss *via* the leaf stomata, we first investigated cumulative water loss from shoots of intact plants grown in pots in potting mix. WT and *AtTOR* expressing plants were grown under well-watered conditions for 50 DAG, and just before the watering was withheld for the subsequent 15 days, the pot containing the intact plant in potting mix was weighed for each replicate plant for each genotype. Subsequently, the plants in pots were weighed at days 4, 8, 12, and 15 of the drought period, and as shown in Figures 3*B* and S3, transpirational water loss was significantly reduced throughout the drought-challenged conditions when *AtTOR* expression was driven by either the 35S or GC-specific promoter in *AtTOR* transgenic plants, compared to the WT controls. In Figure 3*B*, transpirational water loss was measured as the loss of the weight of water in the pots over the 14-drought period, while in Figure S3, transpirational water loss was measured as the % of the maximum soil water content, which was 60% of the full water holding capacity of the soil in pots under well-watered conditions. Subsequently, we also measured the water loss of detached leaves from the three genotypes over an 8-h period after the leaf was detached from the plant (Fig. 3*C*). For both transgenic lines, water loss from detached leaves was significantly less than for leaves from WT plants during the 8-h leaf dehydration period. These results indicate that improved performance in response to drought in *AtTOR* overexpressing lines is associated with reduced transpirational water loss from the leaves and suggests that ectopic expression of *AtTOR* in guard cells (Fig. 2*C*) will confer improved water conservation compared to the control in terms of leaf water vapor fluxes and loss through the leaf stomata.

### Measurements of leaf stomatal physiology revealed improved photosynthetic performance and reduced water loss in *AtTOR* overexpressing plants

**Figure 4:**
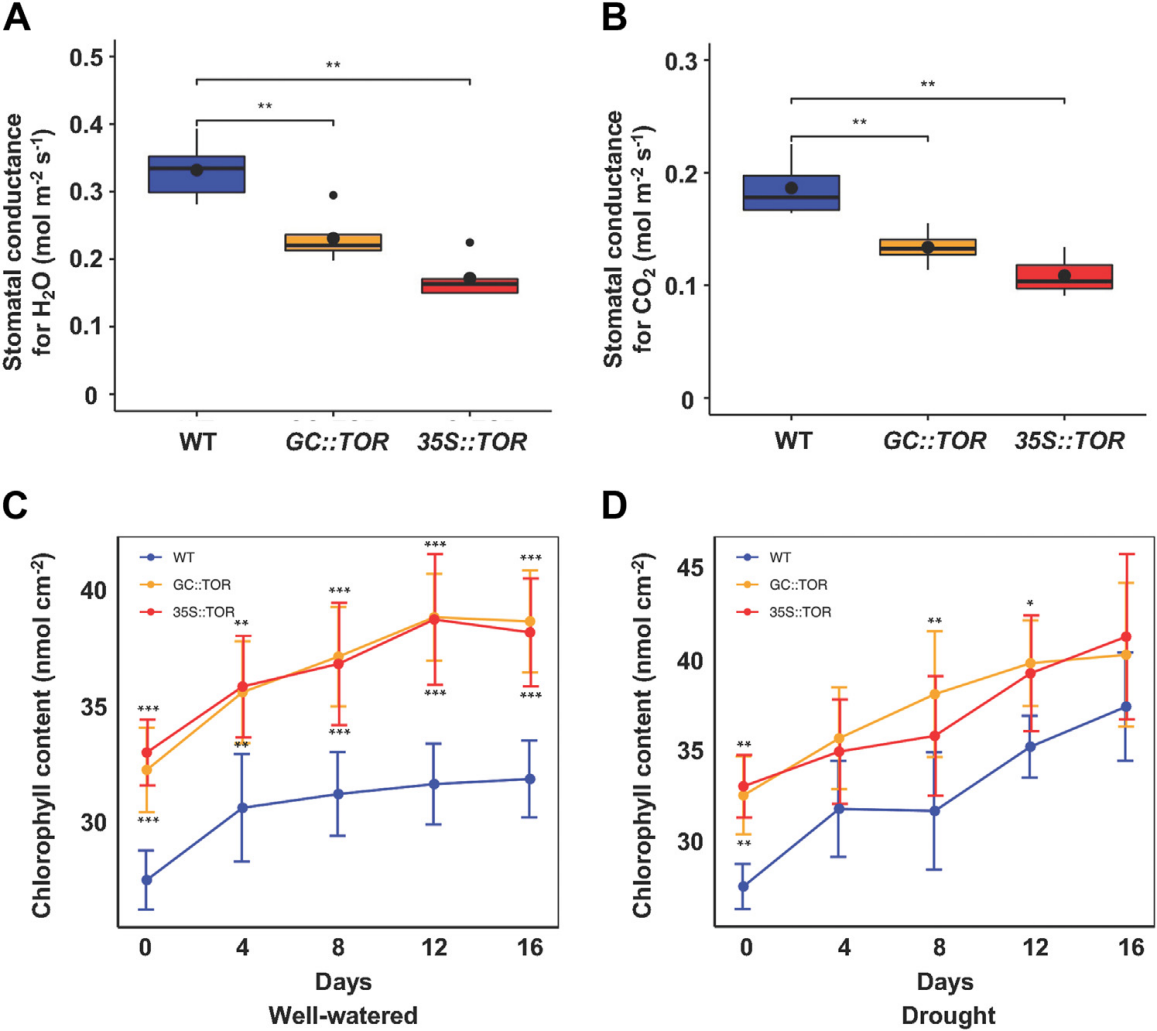
**Leaf gas exchange properties and chlorophyll concentrations for WT and *AtTOR* transgenic lines.***A*, stomatal conductance to water (GasEX_gsw), measured at a light intensity of 1000 μ mol m^−2^ s^−1^ of photosynthetically active radiation (PAR). The stomatal conductance data is depicted as box plots. The *black circles* within each box plot represents the mean value for stomatal H_2_O or CO_2_ conductance and the *black circles* beyond the whiskers represent outliers. Both *TOR* transgenic lines had lower stomatal conductance to water vapor compared with WT plants. *B*, stomatal conductance to CO_2_ (GasEX_gtc), measured under the same conditions as in a. The stomatal CO_2_ conductance for both *AtTOR* transgenic lines were lower than in WT, and the differences are significant. Gas exchange measurements were performed on 50 DAG plants under short-day (8 h light) and well-watered conditions on fully expanded leaves using the LI-COR Biosciences 6800 Photosynthesis system (Lincoln). For all measurements, at least four independent plants were used as biological replicates or each of the 3 genotypes and the experiment was repeated three times. *C* and *D*, measurement of leaf chlorophyll concentrations under well-watered (*C*) and drought stress (*D*) conditions for WT and both *AtTOR* transgenic lines. Assay of leaf chlorophyll concentrations under well-watered and drought conditions was determined using a SPAD-502Plus chlorophyll meter. The plants were initially grown for 50 DAG under short-day (8 h light) and well-watered conditions. Then the chlorophyll measurements were made every 4 days over a 16-day period of drought or well-watered conditions. The comparison of the leaf chlorophyll content between WT plants and both transgenic lines (*35S::AtTOR* and *GC::AtTOR*) is significantly different at most time points where chlorophyll contents are measured. Analysis of variance of the data was conducted using the Student’s *t* test. ∗, 0.01 < *p* < 0.05; ∗∗, 0.001 < *p* < 0.01; ∗∗∗, *p* < 0.001 (top ∗: *GC::AtTOR* lines vs. WT, bottom ∗: *35S::AtTOR* lines vs. WT in *C*, *D*).

*TOR* is closely associated with several growth characteristics in plants, including development of leaf stomata. To investigate whether increased *AtTOR* expression in the transgenic lines contributes to changes in leaf stomatal physiology, we first measured the leaf stomatal density in all three genotypes. The results showed significant differences between WT and transgenic lines. Lower densities of abaxial leaf stomata were measured in *AtTOR* transgenic lines compared to WT plants (Fig. S4). To delve deeper into how the leaf stomatal physiology affects the regulation of stomatal functions and leaf photosynthesis, we conducted infrared gas exchange analysis on Arabidopsis leaves, to quantify the stomatal conductance to water vapor leaving the leaf, CO_2_ flux into the leaf, as well as leaf photosynthetic CO_2_ assimilation (Figs. 4, *A* and *B*; S5). Consistent with our plant and leaf water loss results shown above in Figure 3, *B* and *C*, both transgenic lines had a lower stomatal conductance to water flux out of the leaf compared with WT plants (Fig. 4*A*). One would expect that reduced water loss due to a lower stomatal conductance for water would also result in a lower CO_2_ flux into the leaves, which is what we observed in Figure 4*B*. In comparing *35S::AtTOR* and *GC::AtTOR* transgenic lines, the stomatal conductance to water vapor and CO_2_ in Figure 4, *A* and *B* showed somewhat larger reductions in stomatal conductance to water in the *35S:*:*AtTOR* expressing line in relation to WT (a 60% reduction), compared to the reduction in stomatal water conductance in the *GC::AtTOR* expressing line vs. WT (a 35% reduction). The same trend was seen for stomatal conductance for CO_2_, where similarly sized differences in reductions in stomatal CO_2_ conductance were seen, with moderately larger reductions in CO_2_ conductance between *35S::AtTOR* expression lines and WT compared with *GC::AtTOR* expressing lines vs. WT.

But surprisingly, despite the reduced CO_2_ flux into the leaves, which should result in lower CO_2_ concentrations inside the leaf, *AtTOR* transgenic plants also exhibited significantly higher relative photosynthetic CO_2_ assimilation rates compared to WT (Fig. S5), even when they possessed a lower stomatal conductance for CO_2_ (Fig. 4*B*). In comparing the increased photosynthetic efficiencies in the two ectopic *AtTOR* expressing lines, it was interesting to note that although both lines significantly stimulated photosynthetic CO_2_ assimilation, the *GC::TOR* lines had a much greater stimulation in photosynthetic CO_2_ assimilation (90% increase over WT plants) compared to *35S::TOR* lines (33% increase over WT plants). As all leaf cells participate in photosynthesis, the significant effect of expressing the *TOR* gene only in leaf guard cells to stimulate photosynthesis in the leaf certainly strongly suggests there must be efficient communication and signal between guard cells and the other cells of the leaf. The stimulation of photosynthesis in response to increased ectopic *AtTOR* expression in leaves suggests that the role of *AtTOR* transgene in drought resistance is likely pleiotropic, and involves pathways minimizing water loss due to transpiration, while also being involved in one or more additional pathways compensating for potentially reduced CO_2_ concentrations in the leaf by increasing the photosynthetic assimilation rate and/or increasing the affinity of the RUBISCO enzyme to leaf CO_2_ concentrations within the leaf. The different possible reasons for this pleiotropic effect of high *AtTOR* expression in the whole plant or only in guard cells are considered in the discussion.

Because chlorophyll plays an important role in light reception for energization of photosynthesis, leaf chlorophyll concentrations under well-watered and drought conditions were determined in the 3 genotypes using a SPAD-502 Plus chlorophyll meter. The plants were initially grown for 50 DAG under well-watered conditions, and then separate sets of plants were measured for chlorophyll content over 16 days of drought or well-watered conditions. In both well-watered and drought conditions, leaf chlorophyll content in both *AtTOR* transgenic lines was significantly higher than that in WT to the same magnitude for both lines, which may increase leaf photosynthetic energy capture (Fig. 4, *C* and *D*). We did not observe differences in leaf chlorophyll content between well-watered and drought treatments. Under both of these conditions, both *AtTOR* transgenic lines had similarly greater leaf chlorophyll concentrations than did leaves in WT plants.

**Figure 5:**
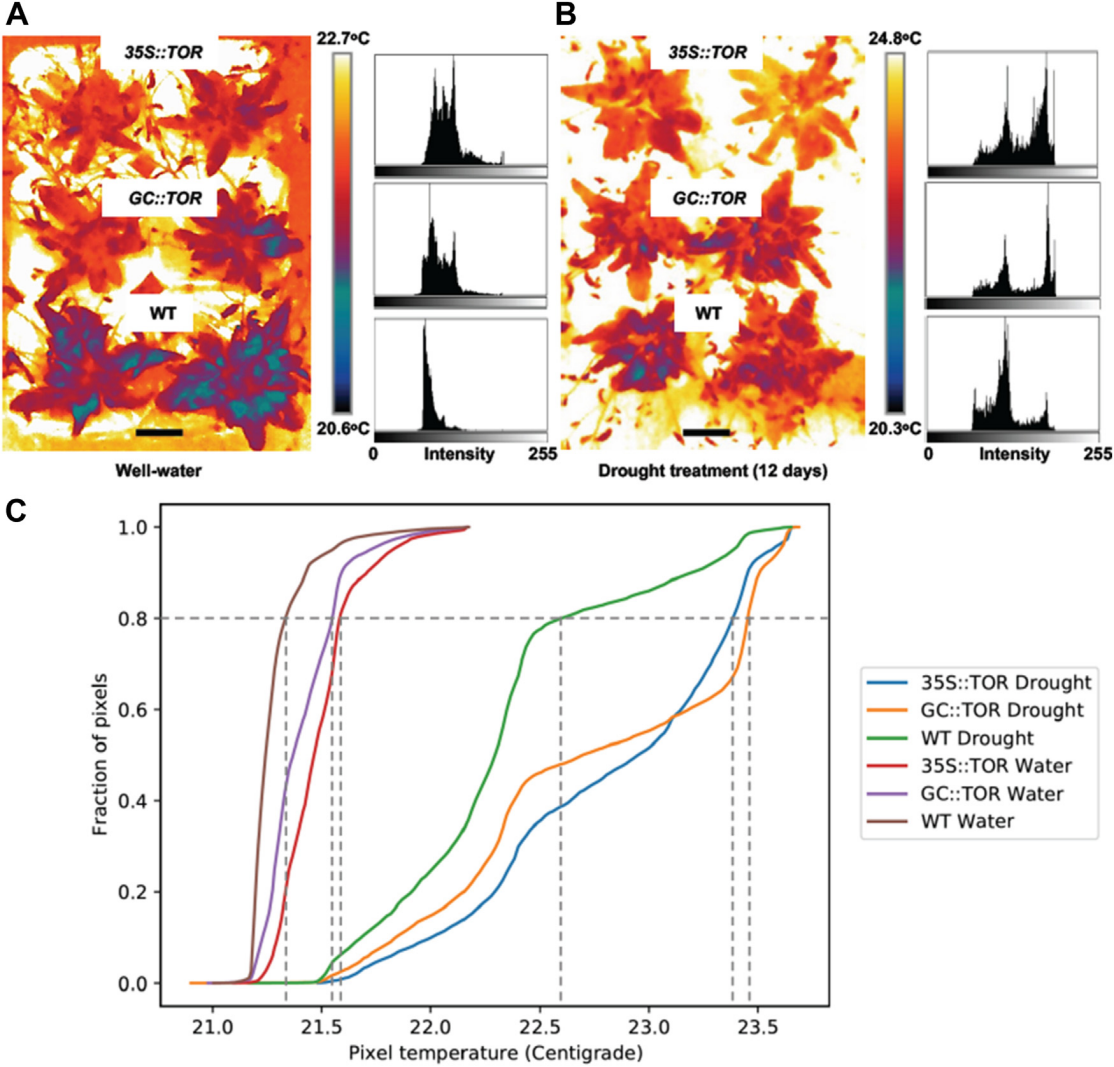
**Canopy temperature in WT and *TOR* transgenic lines measured *via* infrared thermal imaging.***A* and *B*, thermal imagery of the Arabidopsis rosette leaves was carried out using a FLIR A655sc infrared camera (FLIR Systems, Inc). Measurements were taken on entire rosettes of well-watered (*A*) and drought-stressed (*B*) WT, *GC::AtTOR* and *35S::AtTOR* transgenic lines. Four biological replicate plants were used for each experiment. The plants were initially grown for 50 DAG under long-day (16 h light) and well-watered conditions. Then water was withheld from half of the plants and the other half of the plants continued to be well watered for the subsequent 12 days of drought or well-watered conditions. Subsequently, thermal images of the entire rosette were taken, and leaf surface temperatures were calculated using the IMAGEJ (Fiji v.1.51u) thresholding and magic wand tools. Scale bar = 3.0 cm. *C*, graph of the distribution of the fraction of leaf pixels at or below any specific canopy temperature. The measured thermal imagery was obtained using the FLIR Systems proprietary format and then converted to *gray*-scale images which were further processed in Fiji 2 Imagine software to quantify the temperature distribution across the rosettes for WT, *GC::AtTOR* and *35S::AtTOR* transgenic lines. Under both well-watered and drought conditions, the shape of the leaf rosette temperature curve for WT plants was clearly distinguishable from leaf rosette temperature curves for the *GC::AtTOR* and *35S::AtTOR* OE lines, at 12 days of drought treatment. The rosette leaf surface temperatures of the *AtTOR* transgenic lines were considerably higher than in the WT plants, which is consistent with the data in previous figures suggesting the *TOR*-expressing lines lose less water from the leaf stomata. The vertical dotted lines for each genotype’s temperature plot indicate the rosette leaf surface temperature at which 80% of the pixels in each rosette thermal image are at or below that temperature. The different colored traces represent the leaf rosette temperature for each genotype under well-watered or drought conditions. The mean plant canopy temperatures obtained from FLIR tools are depicted in the box to the *right* of the plots of plant leaf rosette temperature.

An indirect outcome associated with leaf evapotranspiration, lower leaf canopy temperature, was measured using infrared thermal imaging to assess how ectopic expression of *AtTOR* affected evaporative cooling *via* transpirational water loss. Under well-watered conditions, the leaf canopy of lines overexpressing *AtTOR* were 0.2 °C warmer than WT at 50 DAG, and after a subsequent 12 days of drought, the transgenic lines’ leaf canopies were 0.75 °C warmer, based on a quantitative analysis of the temperature associated with 80% of the rosette image pixels as determined using IMAGEJ (Fig. 5, *A*–*C*). Higher leaf surface temperatures in the transgenic lines under both conditions are consistent with less evaporative cooling due to reduced transpiration in the transgenic lines compared to the WT (Fig. 5, *A*–*C*). These findings are consistent with all of the above findings, demonstrating that the expression of *AtTOR* in transgenic lines causes greater stomatal closure under drought compared to WT, thus conserving more leaf water under drought, with a reduction in leaf stomatal number also contributing (Fig. S4).

### *AtTOR* ectopic expression improved drought resistance, which involves a significant increase in water use efficiency during the drought stress period

Examination of instantaneous WUE (WUEi), based on the measurement of CO_2_ assimilation and transpiration rate of whole plants, was performed under different light intensities using the LiCOR 6800. The results showed at a light intensity of 900 μmol m^−2^ s^−1^, there was an approximately 50% increase in WUEi in *GC::AtTOR* lines and a 20% increase in *35S::AtTOR* lines over WT with well-watered plants. Because of the greater plant growth in *AtTOR* transgenic lines compared to WT lines under well-watered conditions (Fig. S6*A*), it was necessary to then determine if the transgenic lines had greater WUE during the drought period compared to WT. Hence, the long-term WUE of each genotype was measured over the whole drought treatment assay period. Plants were initially grown for 27 DAG under long-day and well-watered conditions, plant dry biomass data were collected at the end of the well-watered period, and then at the end of the 3 subsequent time intervals during the 11 day period when drought treatment was imposed, and also at the end of the subsequent 4-days recovery period, when water was again provided to the drought-stressed plants (Fig. S6, *A*–*E*). In this figure, it is clear that the two *AtTOR* transgenic lines generate more shoot biomass than the WT Arabidopsis plants, especially at the latest drought period (drought on days 9–11) and during the 4-day recovery from the drought period due to rewatering.

**Figure 6:**
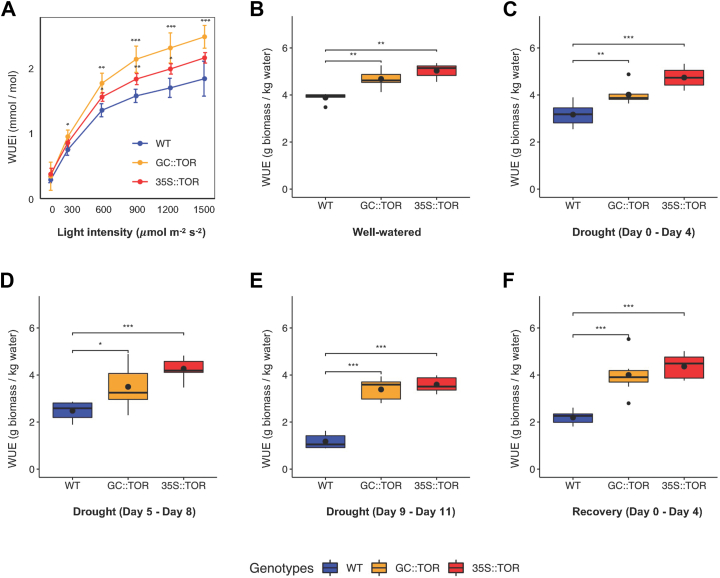
**Water use efficiency in WT and *AtTOR* transgenic lines.***A*, instantaneous water use efficiency (WUEi) in WT and *AtTOR* transgenic lines under different light intensities. Light response curves (at 150, 300, 600, 900, 1200, 1500 μmol m^−2^ s^−2^) were established for WUEi in WT, *35S::AtTOR* and *GC::AtTOR* lines. All data were collected using plants grown at 27 DAG using the LiCOR 6800 system with the small plant chamber under well-watered condition. X-axis, light intensity; Y-axis, WUEi measurement (A/E) from LiCOR 6800 system. Standard derivations were calculated for each light intensity between WT and *AtTOR* transgenic lines. *B*, boxplots of long-term water use efficiency (WUE) which was calculated based on the increment of biomass produced and water transpired during the 8-days time period (19–27 DAG) under well-watered conditions. *C*–*E*, boxplots of long-term WUE which was calculated based on the increment of biomass produced and water transpired during the specific period of drought. Water was withheld on 27 DAG. *C*, long-term WUE was calculated for the first 4 days of drought treatment (27–31 DAG). *D*, long-term WUE was calculated for the next 4 days of drought treatment (31–35 DAG). *E*, long term WUE was calculated for the last 3 days of drought treatment (35–38 DAG). Plants were then provided with water again on 38 DAG. *F*, boxplots of long-term WUE calculated based on the increment of biomass produced and water transpired during the 4-day period of rewatering after the drought period (39–42 DAG). Five biologically replicate plants were used for each experiment. The *black circles* within the box of each box plot represents the mean value for long term water use efficiency and the *black* circles beyond the whiskers represent outliers. ∗, 0.01 < *p* < 0.05; ∗∗, 0.001 < *p* < 0.01; ∗∗∗, *p* < 0.001.

Subsequently, as depicted in Figure 6, *B*–*F*, the long-term WUE of both *AtTOR* transgene expressing lines was significantly higher than in WT plants, and the differences in WUE were greatest during the drought and recovery periods. The maximum increase in long-term WUE in *AtTOR* transgenic lines was 50% higher than WT plants after 4 days of drought, 160% higher than WT plants after 11 days of drought, and 110% higher than WT plants after 4 days of recovery from drought. (Fig. 6, *C*–*E*). This demonstrates that significantly improved WUE (photosynthetic CO_2_ assimilation per unit water lost *via* evapotranspiration) under both well-watered and especially drought conditions is associated with the ectopic expression of *AtTOR* in guard cells. These findings, along with the measured increase in photosynthetic CO_2_ assimilation in both *AtTOR* transgenic lines during these time periods, suggest that the transgenic plants confer drought resistance by reducing water loss during drought, but also experience increased biomass production likely mediated through enhanced photosynthetic performance, possibly *via* a different TOR-mediated pathway. It is also interesting to note that the WUE in well-watered transgenic plants (Fig. 4*B*) was relatively close to the WUE in these same transgenic *AtTOR* lines under drought and recovery from drought. These results strongly suggest the possibility that we might be able to increase plant WUE *via* manipulation of At*TOR* expression, with both significant decreases in transpirational water loss and significantly higher photosynthetic performance under drought compared to WT plants.

### *35S::AtTOR*, and *GC::AtTOR* lines had higher TOR protein expression and TOR activity (S6K1 phosphorylation) after drought treatment

**Figure 7:**
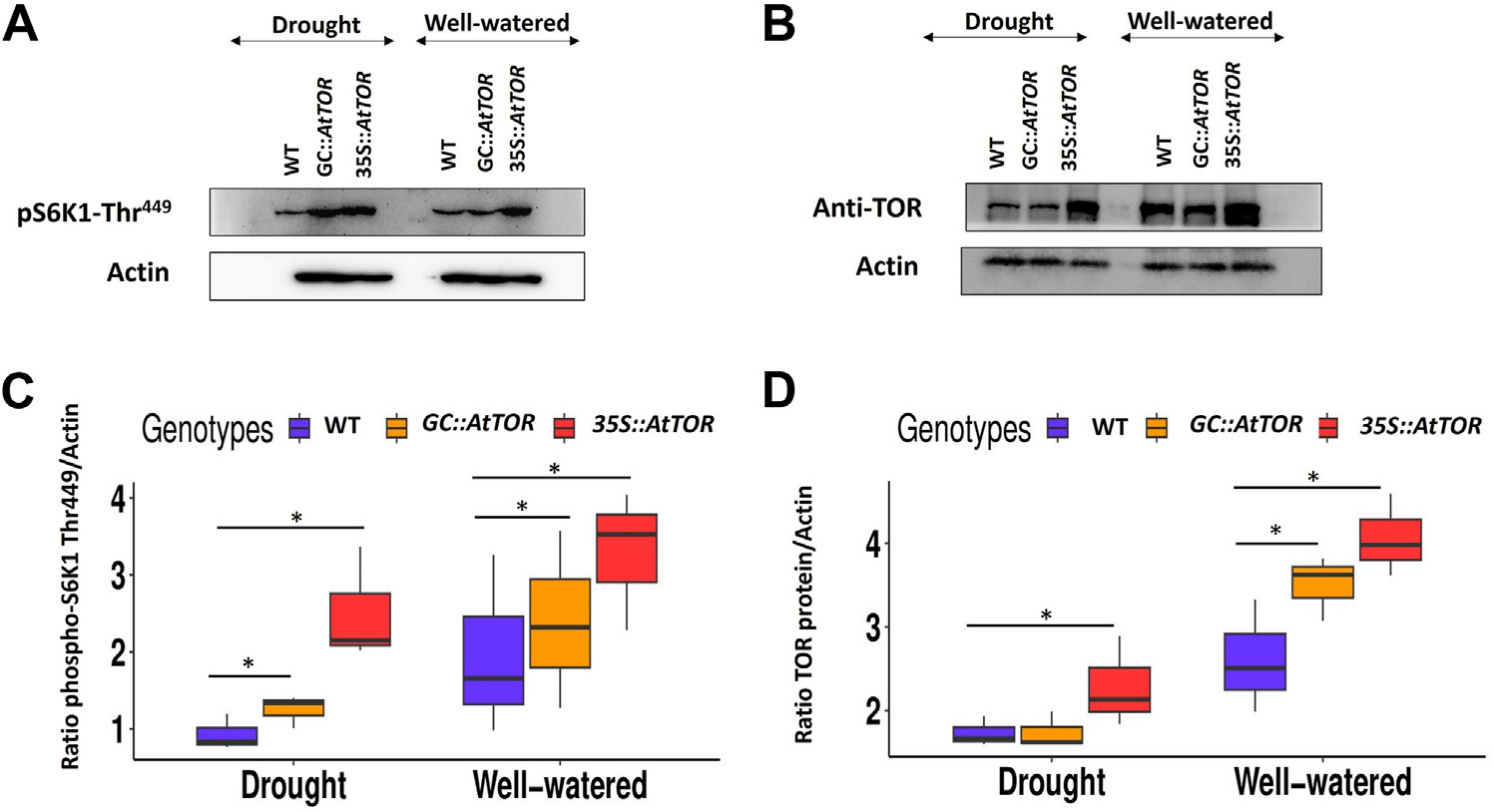
**TOR expression and TOR activity (S6K1 phosphorylation) under drought stress.** The *35S::AtTOR* and *GC::AtTOR* lines had higher TOR expression and TOR kinase activity (S6K1 Phosphorylation) compared to the WT plants under drought stress. *A*, S6K1 phosphorylation was detected using phospho-anti-70S6K1-Thr(P)^449^ (Catalog# ab207399, Abcam). Additionally, the 35S::AtTOR lines showed significantly higher TOR protein levels and TOR activity (S6K1 Phosphorylation at Threonine-449 residue) under both drought and well-watered conditions compared to the WT and GC::AtTOR lines (and GC::AtTOR lines had higher TOR kinase activity than wild type plants in response to well-watered and drought conditions). *B*, TOR protein expression level in WT (Col-0), GC::AtTOR, 35S::AtTOR lines under drought and well-watered conditions were analyzed using the anti-TOR antibody (Catalog# AS12 2608, Agrisera), and (*C* and *D*) Quantification of intensity of signals from S6K1 phosphorylation and TOR expression normalized and represented as the ratio with Actin (Catalog# ab197345, Abcam). The data shown in the graphs (*C* and *D*) is represented as means of three independent biological replicates with ± standard error. One-way ANOVA was performed and significant values at *p* < 0.05 are represented with asterisks “∗”.

To assess whether the drought resistance phenotypes observed and quantified in the *AtTOR* transgenic lines are associated with enhanced TOR protein expression and enzyme activity in addition to the increased *TOR* transcript levels, we have used the well-established 70 kDa ribosomal S6 kinase 1 (S6K1)-based assay to measure TOR kinase activity. This assay is based on the phosphorylation of the 449th Threonine residue in TOR’s hydrophobic motif by mTORC1, the target of rapamycin complex (27). To quantify TOR activity, we analyzed S6K1 phosphorylation levels, in the *35S*::*AtTOR*, *GC*::*AtTOR* and WT lines under drought stress and well-watered conditions. Both *35S::AtTOR* and *GC::AtTOR* lines had significantly higher TOR kinase activity under drought compared to WT plants (Fig. 7, *A* and *C*). We also used Western blot analysis to show that the *35S*::*AtTOR* line had higher TOR protein abundance under drought conditions compared to WT, which as expected, was higher than in the *GC::AtTOR* line, because in guard cell *TOR* expression, only a tiny fraction of the leaf cells are expressing the *TOR* gene and presumably protein to high levels (Fig. 7, *B* and *D*). Hence, it is interesting that the *GC::AtTOR* expressing line did have significantly higher TOR protein abundance compared to WT under well-watered conditions (Fig. 7*D*). The TOR protein expression levels were reduced after drought stress in all the lines but were higher in the *35S::AtTOR* lines than in WT plants. (Fig. 7, *B* and *D*). To further confirm these observations regarding TOR protein abundance, Western blot analysis was performed using three independent biological replicates, and as shown in Figure S7, *A*–*D*). The results were consistent with higher TOR activity levels and protein abundance in the *AtTOR*-expressing transgenic lines, with the greatest increases coming in the *35S::AtTOR* transgenic lines. Collectively, the results indicated that *AtTOR* constitutive *ectopic* expression and guard cell-specific *AtTOR* expression increased TOR kinase activity under drought stress when compared with WT plants.

### Transcriptome analysis of *AtTOR* transgenic lines indicates that the expression of stomata and ABA-related genes are differentially regulated during drought stress

**Figure 8:**
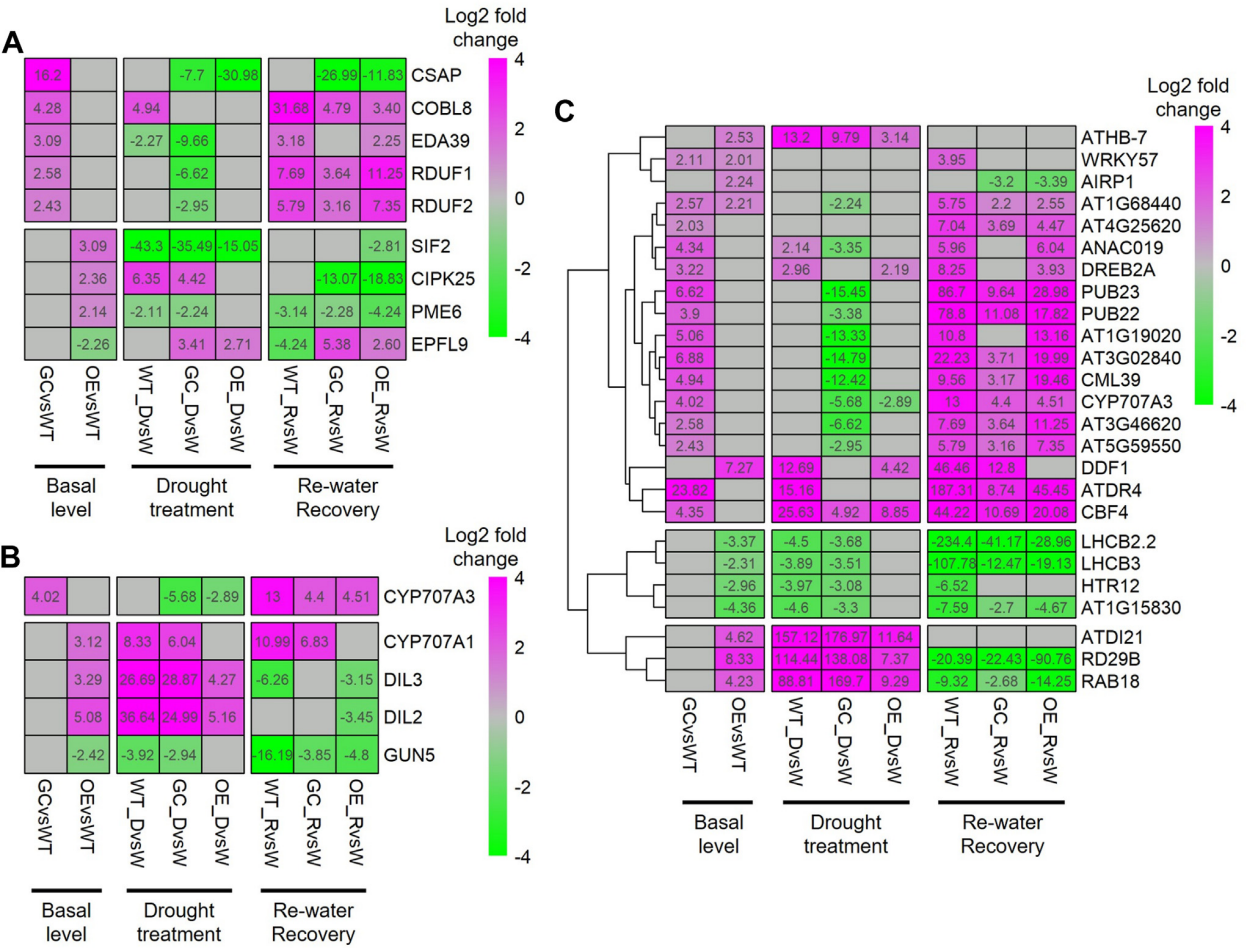
**RNA-seq analysis of differentially expressed genes (DEGs) in the three different genotypes (WT, *GC::At*TOR, and *35S::AtTOR*) in response to the 3 different watering regimes (watered, drought imposed, and subsequent recovery *via* re-watering).** Heatmaps of DEGs from the 3 genotypes quantifying differential gene expression responses in the 3 different watering regimes focusing on: (*A*) stomata-related genes, (*B*) ABA-related genes, and (*C*) other drought-related genes that were identified. DEGs with similar expression patterns are clustered in (*C*), with three major clusters identified and separated by white borders. Z-score transformations of expression were performed for each gene across all samples under different treatments. Expression levels (log2-transformed fold changes) are indicated by the color scheme, from *magenta* (high differential expression) to *green* (low differential expression) in the corresponding conditions, as defined at the *bottom* of the heat map. The fold gene expression change values are labeled for each sample in each cell of the heat map. Three biological replicates were used for RNAseq on each genotype x condition. WT: wild type; GC: *GC::AtTOR*; OE: *35S*::*AtTOR*; W: Well-watered; D: Drought treatment; R: Re-watered recovery.

To begin to elucidate the potential mechanism(s) contributing to differences in leaf stomatal physiology between WT and transgenic lines, we examined the expression of stomata-related genes in response to different conditions by performing genome-wide transcriptome analysis employing RNA-seq on the three Arabidopsis genotypes grown on the three water treatments. Principal component analysis (PCA) clearly separated the samples from the three different treatments into three groups (well-watered, drought treatment and rewatered recovery; Fig. S8). Further analysis of differentially expressed genes (DEGs) revealed that a significant number of these genes had major changes in their expression in response to the applied drought and re-watered recovery conditions in *AtTOR* transgenic plants compared to WT (Figs. 8, *A*–*C*; S9). Under well-watered conditions, several stomata-associated genes are upregulated in the two *AtTOR* expressing lines compared to WT (Fig. 8*A*), including the genes *CSAP*, *COBL8*, *EDA39*, *RDUF1* and *RDUF2*, which are upregulated in *GC::AtTOR* lines; while *SIF2*, *CIPK25* and *PME6* are upregulated in *35S::AtTOR* lines. All of these genes are involved with ABA, ranging in roles including ABA response, ABA sensitivity, ABA suppression, and ABA-induced stomatal movement (28, 29, 30, 31, 32, 33). The only downregulated stomata-related gene in the well-watered *35S::AtTOR* line was *EPFL9*, which is a positive regulator of stomatal development (34). These results suggest that *EPFL9* could likely play a role in the reduced stomatal development we documented in the *AtTOR* expressing transgenic lines (Fig. S4).

Meanwhile, several ABA metabolism-related genes are also differentially regulated in *AtTOR* transgenic plants compared to WT under well-watered conditions (Fig. 8*B*). Two cytochrome P450 enzymes, *CYP707A3* and *CYP707A1* are upregulated in *GC::AtTOR* and *35S::AtTOR*, respectively. *DIL2* and *DIL3*, ABA-induced regulators of ABA signaling, are upregulated in only *35S::AtTOR* transgenic plants. We then extracted all drought response genes from our DEG data set by performing clustering analysis, and identified the genes differentially expressed under well-watered conditions (Fig. 8*C*) or during the drought stress and re-watered recovery periods (Fig. S9). Interestingly, the two *AtTOR* transgenic lines exhibit different regulatory patterns for these genes. Only two genes were upregulated in both *GC::AtTOR* and *35S::AtTOR* transgenic plants, while the other 23 DEGs are only differentially expressed in one of the two AtTOR transgenic lines (Fig. 8*C*). For DEGs under the drought stress and/or rewatered recovery treatments (Fig. S9), at least three major clusters were identified. One cluster of genes was highly expressed during drought treatment (bottom), and another cluster of genes were highly expressed during recovery (top). Meanwhile, in the middle cluster, some drought-related genes were only upregulated in *GC::AtTOR* plants during drought treatment, for example, OST1, MBF1C and MYB32, which have been previously shown to respond to ABA to mediate specific processes, or be involved in stomatal opening and closure (35, 36, 37). Although they are similarly expressed under well-watered conditions in both WT and transgenic plants, their different response to drought in *GC::AtTOR* plants might activate a different adaptive defence mechanism compared to WT and *35S::AtTOR* plants. Taken together, the observed differentially regulated gene activities associated with stomata, ABA and stress response likely play important contributions to the drought resistance responses in *AtTOR* transgenic plants. These identified gene signatures suggest that their respective functions are regulated by TOR signaling pathways, thus providing new insights into key molecular and biochemical processes underpinning drought responses that are integrated with photosynthetic efficiency.

## Discussion

The modulation of growth and development is a central process in all organisms, and an intimate relationship exists between water and nutrient availability, energy status, and cell growth rate, which are influenced by dynamic and diverse environmental stresses and challenges. Hence, growth and development of all eukaryotic organisms is not a simple sum of available energy and nutrients, but instead represents a regulated set of interactions, including responses to external factors that support complex survival and reproductive strategies. Extensive research on a number of eukaryotic organisms, ranging from yeast to humans, has demonstrated that the TOR kinase acts as a master regulator to sense and transduce nutrient and energy status, hormonal levels and their activities, and growth factors and stress inputs, linking these signals to metabolic and biological processes that drive cellular, tissue and organismal growth (19, 38, 39, 40). In plants, TOR signaling has also been demonstrated to play fundamental regulatory roles in hormone signaling, embryogenesis, meristem activation, root and leaf growth, flowering, senescence, and life span determination (15, 16, 17, 41, 42, 43, 44). Recent research has provided more evidence that TOR is involved in plant environmental stress responses, including cold, and osmotic stress (20, 45, 46). Moreover, investigations on the crosstalk between TOR signaling and ABA signaling have revealed additional potential roles for TOR in abiotic stress. Consistent with this, TOR signaling has been found to regulate ABA biosynthesis and distribution (47). Upon sensing environmental stress, plants usually transiently sacrifice growth and activate protective stress responses through reciprocal crosstalk between TOR and ABA signaling (48). Such a trade-off between plant growth and stress adaptation was considered as a Yin and Yang control in which TOR signaling was involved (49). Under growth-promoting conditions, active TOR phosphorylates the ABA receptors, PYR/PYLs, to down-regulate ABA signaling, and directs resources toward growth. Also, through activation of SnRK2s, which allows the phosphorylation of Raptor to dampen TOR activity, which limits growth for survival during stress conditions (50). This regulatory reprogramming provides both balance and dynamic responses between TOR and ABA signaling, in response to certain abiotic stresses. However, it is unclear how exactly the underlying physiological and molecular mechanisms and the associated regulatory networks regulate the long-term water use efficiency under water challenged conditions, such as drought, in which it appears complex TOR functions operate.

In the present study, we show that plants ectopically expressing *AtTOR* result in increased *TOR* transcript and TOR protein expression/activity, exhibit faster shoot growth and the development of a larger root system, along with increased plant biomass (Figs. 2 and 6; S6). More importantly, we demonstrate that *AtTOR* ectopic expression positively regulates growth, development and survival in response to water limiting conditions and results in rapid recovery from drought when water is re-introduced to the plant. We have expressed *AtTOR* using either a constitutive 35S promoter or a guard cell-specific promoter and found that each promoter driving *AtTOR* ectopic expression is able to confer resistance to drought, as well as having positive growth effects under water-limited conditions. The reason we tested the hypothesis that guard cell expression of *AtTOR* would, like 35S driven *AtTOR* expression, confer improved WUE, was because of the absolutely essential roles leaf guard cells play in regulating water vapor and CO_2_ fluxes out of and into leaves, and also in the utilization of these two essential plant resources. Importantly, the 35S promoter activates expression constitutively throughout the plant, including guard cells, and this overlap likely results in a number of similar drought response outcomes in both transgenic lines. Indeed, the guard cell-targeted specific expression of *AtTOR* is sufficient to confer both increased drought resistance in Arabidopsis and enhanced water use efficiency under both well-watered and drought stress conditions. As shown in Figure 3*A* and Movie S1, *GC::AtTOR* transgenic plants have a much greater ability to tolerate a prolonged drought period, with a subsequent rapid recovery from drought upon re-watering, compared to WT plants, which did not recover when re-watered after the same drought period. The fact that we can demonstrate increased drought resistance and also improved WUE under drought using the guard cell-specific promoter suggests that one possible role of *AtTOR* involves the regulation of stomatal functions.

Consistent with this, the transcriptome studies performed in this study revealed activation of gene expression associated with guard cells as well as ABA signaling and general stress response in the *AtTOR* transgenic lines (Figs. 8, S9). A number of genes involved in the regulation of stomatal function are overexpressed in *35S::AtTOR*-expressing transgenic lines compared to WT, including the genes *PME6 (pectin methyl transferase 6)*, *SIF2 (stress-induced factor 2)*, and *CIPK25* (*calcineurin β-like interacting protein kinase 25*). All three of these genes are needed for optimal stomatal functioning, especially under environmental stress. In *GC::AtTOR*-expressing transgenic lines, the genes *RDUF1* and 2 (*E3 ubiquitin-protein ligase*), *EDA39* (*Calmodulin-binding family*), and *COBL8* (*Cobra-like protein 8*) were more highly expressed than in WT plants. *AtRUDF1/2* are involved in the positive regulation of ABA-dependent drought stress responses, *AtEDA39* is involved is stomatal movement, ensuring proper opening and closing of the guard cell pairs making up the individual stomata, and *AtCOBL8* is a key player in stomatal development, regulating the deposition of cellulose in the guard cell wall, so that the stomata can open and close efficiently. Also, we discovered that *35S::AtTOR* and *GC::AtTOR* expressing Arabidopsis plants showed altered expression of several ABA metabolism genes, with two cytochrome P450 enzymes, encoded by *CYP707A3* and *CYP707A1*, upregulated in *GC::AtTOR* lines. *CYP707A3* has been shown to play an important role in determining threshold levels of ABA during dehydration and after rehydration, while *CYP707A1* encodes abscisic acid 8′-hydroxylase, which is important for proper control of seed dormancy and germination in Arabidopsis (51, 52). We have shown here in both *GC::AtTOR* and *35S::AtTOR* expressing Arabidopsis lines that possibly the ectopic expression of *GC*::*AtTOR* in guard cells resulting in high *CYP707A1* expression is involved in the increase in seed production. Identification of these differentially expressed genes provide important new insights into the molecular and biochemical programs and their associated underpinning regulatory networks influencing growth and development, coordinated with TOR signaling in response to drought stress. Therefore, it is not surprising that we see a number of similar Arabidopsis growth and drought responses when expressing the *AtTOR* gene behind these two different promoters, and we also see some different responses when *AtTOR* is expressed in all Arabidopsis cells *vs.* expression in just guard cells.

To summarize, for the genes we have identified in this study whose expression appears to be regulated by *TOR*, we have shown that some of these genes are involved in the control of stomatal development and guard cell movement, and other genes in different aspects of ABA signaling, with a large fraction of those ABA genes specifically involved in regulating drought response. The known function of these genes, whose expression is increased due to *TOR* overexpression, clearly can impact and enhance drought resistance and WUE (8). Guard cell/stomatal-mediated signaling regulates both transpirational water loss and CO_2_ flux into the leaf, and appropriate modulation of stomatal activity might be an effective way to reduce a plant’s water consumption, allowing the plant to better tolerate periods of drought. In rice, ectopic expression of the Arabidopsis *TOR* gene enhances WUE, growth, and yield under water-limiting conditions (20), suggesting that constitutive *AtTOR* expression might alleviate the effect of drought. Besides constitutive *AtTOR* expression, in the current study, the specific expression of *AtTOR* was limited to guard cells, providing a more accurate resource to study the regulation of WUE by *AtTOR* through the manipulation of stomatal physiology and development under drought-challenged conditions.

The measurements of leaf transpirational water loss, both in intact plants (Fig. 3*B*) and excised leaves (Fig. 3*C*) in the *AtTOR* transgenic lines, showed that both GC-specific and *35S*::*AtTOR* overexpression resulted in significantly reduced water loss compared to WT (Fig. 4). This suggests that the ability of *AtTOR* transgenic lines to resist drought affects under water-limiting conditions is due in part to the ability of these plants to control water loss *via* transpiration. This enables the plants to conserve water and allow for continued growth after the drought is broken. The role of TOR in regulating stomatal function is further supported by the fact that the guard cell-specific *AtTOR* lines had similar reductions in water loss compared to WT plants, as did *35S*::*AtTOR* lines (Figs. 3, S3), suggesting that targeted expression of the *AtTOR* gene in guard cells may be an effective strategy for enhancing WUE under drought. Additionally, as seen in Figures S2 and S6, *GC::AtTOR* transgenic lines grew better than WT, again suggesting dual roles for *AtTOR* in enhancing plant WUE *via* transpirational and photosynthetic control.

Transpirational water flux out of the stomata plays a role in evapo-transpirational cooling of leaves. Hence, it is not surprising that the measurements of leaf canopy temperature *via* infrared imaging depicted in Figure 5 demonstrated that under well-watered and drought conditions, the *AtTOR* transgenic lines had higher canopy temperatures than WT plants, since leaf water loss is reduced in the transgenic lines.

Specific measurements of guard cell physiology in this study supported previous whole plant and whole leaf studies with regards to linking drought resistance to reduced leaf water loss (53). Direct measurements of stomatal conductance to water showed that water fluxes out of guard cells were significantly reduced in both of the *AtTOR* transgenic lines compared with WT plants (Fig. 4*A*). The reduced water vapor flux is presumably due to a reduction in the size of the stomatal aperture and the reduced number of stomata per unit leaf area. This could be problematic, as a strategy to enhance drought resistance *via* increased stomatal closure should also result in reduced CO_2_ flux into the leaf, thus possibly reducing photosynthesis. As shown in Figure 4*B*, overexpression of *AtTOR* did indeed result in a decrease in guard cell CO_2_ conductance. However, quite surprisingly and importantly, *AtTOR* overexpression also resulted in a significant increase in relative photosynthetic CO_2_ assimilation (Fig. S5). This suggests that *AtTOR* has pleiotropic effects in the leaf, which involve increased photosynthetic efficiency. At this time, we can think of a number of possible mechanisms associating higher TOR activity with increased photosynthetic efficiency. One involves higher leaf *AtTOR* expression that either directly (*via* phosphorylation) or indirectly increases the abundance and activity of the RubisCO protein and/or other proteins in the chloroplast stroma involved in photosynthetic CO_2_ assimilation. TOR phosphorylation could also possibly change RubisCO’s assembly, protein interactions, structure and/or function, which could increase photosynthetic efficiency by increasing RubisCO’s affinity for CO_2_, thus increasing CO_2_ assimilation at the reduced CO_2_ concentrations in leaves with fewer and more closed stomata. Finally, we found that both *AtTOR* overexpressing lines had similarly higher leaf chlorophyll concentrations (Fig. 4, *C* and *D*) than WT plants under both well-watered and drought conditions. Possibly this contributes to enhanced photosynthetic efficiency, due to greater and/or more efficient photosynthetic energy capture to drive CO_2_ assimilation. Thus, increased *AtTOR* expression could enhance both or either photosynthetic dark or light reactions, possibly working together to increase photosynthetic efficiency. Ultimately, all of these processes may contribute synergistically and/or additively to the increased biomass production using less water and thus increasing WUE under drought in the *AtTOR* lines.

When data for guard cell conductance to water and photosynthetic CO_2_ assimilation in the *AtTOR* transgenic lines are considered together, we see that ectopic expression of *AtTOR* increases the rate of photosynthesis compared to WT under both well-watered and drought stress conditions, while at the same time reducing water efflux out of the leaf *via* the stomata. Additionally, the fact that Arabidopsis *AtTOR* transgenic lines exhibit reduced stomatal conductance and transpiration with no negative effects on their photosynthesis or growth, indicates that modulation of stomatal/guard cells dynamics and development might be an effective strategic approach for increasing water use efficiency, that is, maintaining reasonable plant yields under water limiting conditions.

This is supported by published findings from several other labs. Expression of the ABA receptor in well-watered transgenic wheat reduced transpirational water loss through greater closure of stomata, leading to reduced CO_2_ concentrations in the leaf; yet photosynthetic CO_2_ assimilation stayed the same in the transgenic wheat and WT wheat (8). In these well-watered plants, the reduction in transpirational water loss with no decrease in CO_2_ assimilation in the transgenic lines, resulted in a 25% increase in instantaneous WUE (WUEi) under well-watered conditions and a 25 to 30% increase in long-term WUE under drought. They also showed that transgenic wheat was not more drought resistant in the vegetative stage than the control wheat line, but they quantified smaller reductions in grain weight, but not grain yield, under drought compared to the control wheat line (a null segregant progeny from the transgenic wheat line). In Arabidopsis, a gene from the epidermal patterning factor family, *EPF2*, which regulates guard cell development, was over-expressed in transgenic Arabidopsis and all the studies were done under well-watered conditions (11). In the transgenic *EPF2* over-expressing lines, leaf stomatal density decreased significantly, by as much as 75%, which led to a decrease in stomatal conductance to water by approx. 50% (11). This, in turn, was associated with no change in photosynthesis compared to WT plants, even with the greatly reduced conductance of the stomata to CO_2_. This decrease in water vapor flux out of the leave combined with no change in photosynthetic CO_2_ assimilation resulted in a 30% increase in instantaneous water use efficiency, but there was no statistically significant increase in time-dependent WUE compared to WT Arabidopsis.

We would like to note that in comparing the findings from these two papers with the results in this manuscript, that it might be difficult to compare Arabidopsis water relations and photosynthetic physiology with a crop species like wheat. But based on the comparison, we did observe similar magnitudes of decreased transpirational water loss, while our WUEi increase compared to WT Arabidopsis grown under well-watered conditions was twice that measured in transgenic wheat grown compared to the null line. That is, there was a 50% increase in transgenic Arabidopsis WUEi over WT, while in wheat, there was a 25% increase in WUEi in transgenic wheat over the null line. Both studies quantified time-dependent WUE under drought, with the transgenic wheat again exhibiting a 25% increase in WUE over the null. However, in the *AtTOR*-expressing Arabidopsis where WUE was measured over an 11-day drought period followed by a 4-day recovery due to rewatering, we observed that maximum increases in long-term WUE in the *AtTOR* transgenic lines were 50% higher than WT plants after 4 days of drought, 160% higher than WT plants after 11 days of drought, and 110% higher than WT plants after 4 days of recovery from drought (Fig. 6, *C*–*E*). The *AtTOR*-expressing Arabidopsis lines were quite drought resistant in the vegetative stage when the long-term drought was imposed, and then during recovery from drought, 100% of the *GC::AtTOR* and 67% of the *35S::AtTOR* plants recovered from drought, flowered, and produced a significant number of seeds. None of the WT Arabidopsis plants recovered from the drought stress. In the study with the ABA receptor-expressing transgenic wheat (8), the transgenic and null lines had the same vegetative biomass production, and there was no difference in seed yield under drought in the transgenic vs. null wheat lines. However, they measured higher seed WUE under drought in the transgenic lines, and although there was no increase in total seed yield, the seed from the transgenic wheat was considerably larger than the null line seed and had a biochemical composition that indicated better wheat seed quality under drought in the transgenic wheat. Finally, they also found small increases in photosynthetic CO_2_ assimilation under drought in the ABA receptor OE line compared with WT, even with the greatly reduced CO_2_ fluxes into the leaf, which likely underlie the increased WUE in the transgenic wheat.

In the study with transgenic Arabidopsis overexpressing *EPF2*, the study was conducted only under well-watered conditions, so no drought resistance data were available. The WUE results were variable, ranging from so significant difference for time-dependent WUE and a 25% difference in WUEi. We have already documented our larger increases in WUEi and WUE over WT plants in the above comparison with reference (8) in wheat. The very large decrease in stomatal density (approx. 75% compared with WT Arabidopsis) translated into a 50% decrease in stomatal conductance to water vapor and presumably CO_2_, which is very similar to the reduction in stomatal conductance in our two ectopic *AtTOR* transgenic lines. As noted above, despite the significant decrease in stomatal conductance to CO_2_ in (11), they saw no decrease in photosynthetic potential and speculated that in the transgenic Arabidopsis lines overexpressing EPF2, thus dramatically reducing stomatal density, stomatal function might be decoupled from photosynthesis. In the current study, we found that despite similar decreases in stomatal conductance to those in (11), we measured a 90% increase in photosynthetic CO_2_ assimilation in the *GC::TOR* expressing line, and a 35% increase in photosynthetic CO_2_ assimilation over WT Arabidopsis in the *35S::AtTOR* line.

We showed here that ectopic expression of *AtTOR* can regulate leaf water loss under water-limiting conditions through a combination of regulation of transpirational water loss through stomata and other, yet to be identified processes that result in increased photosynthesis when partial closure of stomata occurs. The positive effect of overexpressing *AtTOR* on plant growth during water-limiting conditions is a somewhat larger effect under high *AtTOR* ectopic expression throughout the plant compared to the selective expression in guard cells. However, both transgenic lines had very similar levels of reduced transpirational water loss compared to WT plants (Figs. 3; S3) However, given the broadly pleiotropic nature of the TOR gene functions, it may be more likely that higher levels *of AtTOR* ectopic expression in many plant tissues may exert currently unidentified and complex regulatory responses in diverse pathways, some of which might lead to negative effects on plant performance and yield.

The observation that guard cell-specific *AtTOR* expression is associated with increased photosynthetic performance (CO_2_ assimilation of) presumably in the whole leaf (Fig. S5), is quite interesting, as the guard cells make up only a small fraction of the photosynthetically active cells in the leaf. This suggests that *AtTOR* functioning in the guard cell may be associated with the regulation of downstream signaling pathways that are transmitted from the guard cell to other photosynthetically active leaf cells. This is a bit puzzling, as in mature leaves, guard cells are usually symplastically isolated from the adjoining leaf cells, as the plasmodesmata between the guard cells and adjoining cells (54), are blocked by callose or cell wall material. Thus, the transmission of such a signal from guard cells to other leaf cells must be apoplastic, possibly due to efflux of an unknown signal across the guard cell plasma membrane. Hence, the mechanistic basis underlying this increase in leaf photosynthetic activity *via* expression of *AtTOR* only in guard cells is a conundrum, whose resolution awaits further investigation.

In summary, the guard cell-expressing *AtTOR* lines generated in this study displayed reduced transpiration rates compared to WT plants in response to drought stress, which helped maintain leaf/shoot water content for an extended period under drought. At the same time, *AtTOR* ectopic expression in the guard cell leading to reduced stomatal efflux of water did not compromise CO_2_ assimilation and photosynthetic performance, even though CO_2_ flux *via* stomata into the leaf was reduced. The reduced transpiration rates and uncompromised (or less compromised) photosynthetic performance result in significantly enhanced WUE under drought-challenged conditions, and this occurs even under the manipulation of *AtTOR’*s specific expression in guard cells instead of constitutive expression throughout the plant. These results contribute to a new perspective on the importance of identifying the underlying components linked to plant *TOR* function in response to abiotic stresses, focusing in this case on drought stress, and *AtTOR* expression’s impact on guard cell function in the regulation of long-term water use efficiency. Additionally, the combination of RNAseq analysis of differentially expressed genes in *TOR* overexpression lines compared with WT lines under drought identified many extremely interesting genes. These include genes involved in stomatal movement and development, ABA signaling, ABA synthesis, and ABA regulation of drought response, and optimal functioning of the guard cells under environmental stress. This sets us up well for future studies that will enable us to start identifying the complex pathways by which TOR enhances plant drought resistance and improves WUE. Furthermore, the findings presented here suggest that manipulating *AtTOR* gene expression in guard cells *via* transgenic and/or advanced molecular plant breeding approaches may be a powerful strategy for developing crops with enhanced drought resistance as well as higher water use efficiency.

## Experimental procedures

### Generation of transgenic lines

The full-length *AtTOR* (7.4 kb) coding sequence was amplified from an *A. thaliana* (Columbia-0) cDNA library using gene specific primers with the *NotI* restriction site added to 5′ end of the forward primers and the *XmaI* site added to 3′ end of the reverse primers. The full-length *AtTOR* amplified product was then cloned into a TA cloning vector, pCR2.1-TOPO (Invitrogen), and the sequence of this *TOR* cDNA was confirmed by ABI Sanger sequencing. The recombinant clone was digested with *NotI* and *XmaI* and the resulting TOR cDNA fragment was introduced into the p8GWN entry vector (Gateway system). The guard cell-specific promoter was identified by promoter tagging (24) in *Nicotiana tabacum*, and this promoter was found to be located in the *Ntab-TN90*_scaffold3543, 2kb upstream from the putative initiation codon, ATG, of gene LOC107774257. The guard cell and the CaMV 35S promoters were then introduced separately into the recombinant vector to drive full-length *AtTOR* gene expression. The whole insertions, including promoters and *AtTOR* full-length CDS from the p8GWN vector were recombined into the pEarleyGate binary vectors through LR recombination reactions in *E. coli*.

The resulting recombinant expression constructs (Fig. S1*A*) were used to transform WT Arabidopsis plants (Columbia-0) using the Agrobacterium-mediated floral dip method (25). This is a simple and effective transformation method that has exclusively been used for Arabidopsis transformation for many years, in which a number of developing flowers on the Arabidopsis plant are transformed *via* floral dip transformation with Agrobacterium carrying the *AtTOR* transgene.

Molecular validation of positive transformants was performed using specific primers designed for the *AtTOR* gene and its promoter sequences (Table S1 and Fig. S1*B*). Four of the homozygous CaMV 35S lines and four of the homozygous guard cell-specific transgenic lines from T2 generations were further identified through progeny testing, and the derivative homozygous lines in T3 generations were used for developmental and physiological analyses. As no statistically significant difference can be found among the homozygous transgenic plants from the same construct in all analyses conducted in this study, we combined the results of all transgenic lines from the same construct together to increase statistical power and named all homozygous transgenic plants from the two constructs as *35S::AtTOR* and *GC::AtTOR* throughout the study presented here.

### Plant growth conditions

Arabidopsis seeds were surface sterilized in 70% (vol/vol) ethanol for 2 min and then washed three times in sterile deionized water. Sterilized seeds were incubated in 1.5 ml centrifuge tubes with clean sterile-deionized water at 4 to 8 °C for 3 days. Vernalized seeds were spread on half MS growth medium in agar (Murashige and Skoog [MS] Basal medium, 0.22% MS salts; 1% Sucrose; 0.8% plant agar) with 150 μg/ml of the antibiotic, timentin. Transformed lines were selected for resistance to 10 μg/ml of the herbicide, PPT (phosphinothricin). Arabidopsis plants were individually grown in plastic 4" pots (4–1/2" sq x 4" deep) in reach-in growth chambers with long-day (16 h 22 °C: 8 h 20 °C, light: dark cycle) or short-day (8 h 22 °C: 16 h 20 °C, light: dark cycle) conditions. Long-day growth conditions were used for Agrobacterium-mediated plant transformation and for whole plant phenotypic analyses where we could visually compare leaf, floral stem and roots of WT and *AtTOR* transgenic lines. Short-day growth conditions were used to allow longer vegetative plant growth which allow us to conduct the different physiological experiments at the same developmental stages for both WT and transgenic plants (26). Short-day growth conditions were employed for the following experiments: (a) drought resistance (quantification of shoot biomass under well-watered and drought conditions, and subsequent recovery from drought); (b) plant evapo-transpirational water loss under drought in both intact plants grown in potting mix and excised leaves; (c) leaf area analysis and leaf chlorophyll content under drought and well-watered conditions; and (d) measurement of Arabidopsis leaf surface temperatures under drought and well-watered conditions.

### Leaf area analysis

The rosette leaf area of WT and transgenic lines expressing *GC::AtTOR* and *35S::AtTOR*, were measured manually using 35 DAG (Days After Germination) grown plants, by excising every leaf where it emerged, flattening the leaf, drawing the outline of the leaf on 2 mm × 2 mm graph paper, and calculating the area for each leaf. For each line, 10 replicate plants were measured.

### Measurement of transpirational water loss in intact plants

To measure the amount of water lost through the Arabidopsis leaf during drought treatment, 4" pots were filled with 50 g of Sunshine Mix number 3 (Sungro Horticultures). The growth mix was watered to 100% field capacity and after subtracting the pot weight and the potting mix weight, the weight of the maximum water per pot was determined (around 200 g). There were two types of experiments carried out to measure water loss. In the first experiment, two plants were grown per pot, and the potting mix surface in the pots was not covered with plastic film. In the second experiments, one plant was grown per pot, and the potting mix surface in the pot was covered with plastic film to reduce surface evaporation. Both types of experiments yielded similar differences between *AtTOR* transgenic and WT lines, indicating that water evaporation from the potting mix surface was much smaller than leaf transpirational water loss, so only results from plants without the plastic film covering the soil are presented. After 50 days of growth under well-watered conditions (with daily re-watering), all pots were set to contain the same amount of water (120 g per pot), which was approximately 60% of the full water holding capacity of the potting mix in pots which was found to represent well-watered growth conditions based on plant growth and appearance. Then, the Arabidopsis plants were subjected to drought stress by withholding water on half the plants for 14 days. Plants were re-watered for the final 4 days of the experiment. Arabidopsis plants in pots were weighed daily starting with the first day of withholding water. The soil water content was calculated by 120 g minus the difference of pot weight during the drought treatment. The leaf water loss was estimated using the soil water content difference. For each line, five replicate plants were measured. Experiments were repeated 3 times.

### Measurement of leaf transpirational water loss from excised leaves under drought stress

The weight of each of 60 small plastic containers was determined. Then, leaves from individual plants for each of the three genotypes that were watered for 50 days were excised from plants, and placed in the small plastic containers (two leaves per container, leaves abaxial side up). Containers were weighed every 30 min for 8 h. The experiments were repeated three times. At least five independent plants were used for each transgenic line. Percent leaf water loss = (1- the leaf weight measured at each time point/the leaf weighed just after it was excised) × 100.

### Measurement of leaf chlorophyll concentration

A Konica Minolta Chlorophyll Meter SPAD-502Plus was used to collect instant and non-destructive on-site measurements of leaf chlorophyll in well-watered and drought stressed plants. The SPAD-502Plus quantifies the amount of chlorophyll by measuring the absorbance of the leaf in two wavelength regions (red 653 nm; near infrared 931 nm). SPAD readings = log (% transmission 931 nm/% transmission 653 nm). SPAD chlorophyll measurements were made by clamping the SPAD chlorophyll sensor unit on the leaf until the chlorophyll reading was generated. Five fully open mature leaves for five independent plants were used for each genotype. Measurements for each plant were performed with four technical replicates at the indicated time points on well-watered and drought-stressed plants.

### Measurement of leaf canopy temperature

This experiment was conducted using well-watered and drought treated plants (14 days of drought) for WT, *GC::AtTOR* (GC) and *35S::AtTOR* (OE) Arabidopsis lines grown under short-day conditions. All plants were grown under well-watered conditions for 50 DAG at which time thermal imaging of the rosettes was carried out. Then water was withheld from the plants for a total of 14 days and thermal images of the leaf rosettes were acquired at 12 days into the 14-days drought period. Arabidopsis canopy temperature was measured *via* thermal imagery using the infrared camera, FLIR A655sc (FLIR Systems, Inc.), with a sensor size of 640 × 480 pixels, and a spectral range of 7.5 to 13.0 μm. The accuracy of infrared camera was ±2% of the reading. The measured thermal imagery of the entire Arabidopsis plant rosette was obtained in the FLIR Systems’ proprietary format and then converted to gray-scale images which were further processed using Fiji 2 Imagine software, 2009 (ImageJ developers, open-source).

### Measurement of leaf stomatal water and CO_2_ fluxes

Gas exchange measurements were performed on fully expanded plant leaves (grown 50 DAG) using the LI-COR Biosciences 6800 Photosynthesis system with the 6 cm^2^ chamber (Lincoln, Nebraska, USA) at a photosynthetically active radiation (PAR) of 400 to 700 nm, measured between 09:00 to 11:30 AM with a 06:00 AM light cycle start time. The Li-COR leaf chamber conditions were light intensity between 150 and 1500 μmol m^−2^ s^−1^; humidity = 60%; leaf temperature = 25 °C; gas flow = 700 μmol s^−1^ and CO_2_ concentration = 400 ppm. At least four independent plants were used for each of the 3 genotypes, and the experiment was repeated three times. The gas exchange variables, including stomatal conductance to water vapor (gsw) and total conductance to CO_2_ (gtc), were recorded.

### Measurement of water use efficiency (WUE)

Two types of WUE were measured in this study: long-term WUE and instantaneous water use efficiency (WUEi). For the long-term WUE measurement, *AtTOR* transgenic and WT plants were grown in 4-inch diameter pots, each with the same amounts of soil and added water. To reduce evaporative water loss from the soil surface to a negligible level, the soil surface was covered with plastic film, except for a small opening in the plastic for the emerging plants. Plants were subjected to either well-watered or drought conditions in a growth chamber. For both well-watered or drought-stressed plants, the increment of shoot biomass increases and consumed water were recorded over specific time intervals, and at least five plants were sacrificed to measure the plant dry weight at each time point. Long-term WUE was calculated by dividing the increase in plant dry weight over the period of growth by the amount of water consumed during that same time period (WUE in units of g biomass/kg water consumed). Under well-watered conditions, the consumed water was replenished to maintain the soil water content at a constant level for the whole process of assay (approx. 60% of full water holding capacity). Under drought challenged conditions, water was withheld for the 11-day time period between 27 DAG until 38 DAG, the next 4 days of drought (31–35 DAG), the last 3 days of drought (36–38 DAG). Subsequently, the plants were rewatered for 4 days and long-term WUE measured at the end of the recovery period. For measuring plant biomass, the aerial part of each plant was excised, and then dry weight was determined after placing plant samples in a drying oven (VWR international) for 2 days at 80 °C. For measurement of WUEi, the LI-COR Biosciences 6800 Photosynthesis system with the small plant chamber (Lincoln) was used, and measurements were performed using the whole plants at 27 DAG under well-watered conditions, using the settings described above. The light response curve was set, and plants were measured at the light intensities between 150 and 1500 μmol m^−2^ s^−1^. The values for GasEX A (photosynthetic assimilation) and E (transpiration rate) were recorded and WUEi was calculated from A/E for each plant. Five replicate plants were used for each genotype.

### RNA-seq analysis and generation of gene expression profiles

For RNA-seq gene expression profiling analysis, 27 samples from the three treatments (well-watered, drought-treated, and re-watered) of the three genotypes (WT, GC, OE), with three replicates for each condition and genotype, were collected. mRNA-seq libraries were prepared using the Illumina TruSeq UD V1.5 Kit. Libraries were prepared by using RNA according to the manufacturer's instructions and then loaded on an Illumina NovaSeq 6000 platform for pair-end sequencing with 150 cycles. Obtained RNA-seq reads were first preprocessed by trimming the adaptor sequences, filtering low-quality reads, and eliminating short reads using Trimmomatic (55). Then, we used STAR (56) for fast and accurate alignment of cleaned reads to the *A. thaliana* TAIR10 reference genome. Read quantification was performed using the Salmon tool (57) and was further transformed to transcript per million (TPM). To summarize expression levels from the transcript level to the gene level, we used the R tximport package (58) to process the TPM values obtained from Salmon. Expression matrices between genes and the 27 samples were then generated. The expression matrices were transformed to normalized counts through DESeq2 (59) with the tximport-to-DESeq2 approach.

Relative relatedness and reproducibility among biological replicates were examined by principal component analysis (PCA) using the R FactoMineR package (60) A global comparison of the relative relatedness of all 27 RNA-seq samples from different conditions was performed using PCA based on the VST values of expressed genes defined above. Bi-plots of PC1 and PC2 were plotted using the R ggplot2 package (https://cran.r-project.org/web/packages/ggplot2), and the explained variance of each principal component was calculated using the get_eigen value function in the R FactoMineR package.

Expressed genes were determined based on the global expression of each gene across all conditions in which it was expressed with more than 1 TPM in at least one condition. Differentially expressed genes (DEGs, defined as log_2_ fold change >1 or < −1, adjusted *p*-value <0.05) analysis was performed by DESeq2 to compare the individual genes between different conditions.

### Droplet Digital PCR (ddPCR) assay

Primers and probes used for measuring *TOR* gene expression *via* digital drop PCR (ddPCR) were designed to confer specificity to *AtTOR* and for the reference gene, *Actin 2*, as an internal control. An intron-spanning feature was also included in primer design to avoid off-target binding to potential genomic DNA contaminants. Probes were 5′-labeled with 6-carboxyfluo rescein (6-FAM) or 6-carboxy-2, 4, 4, 5, 7, 7 hexachlorofluorescein succinimidyl ester (6-HEX) as the reporter, and 3′-labeled with ZEN and Iowa Black FQ as the double quenchers (Integrated DNA Technologies). Primer and probe sequences are provided in Table S1.

The Arabidopsis genotypes WT, *GC*::*AtTOR*, and *35S::AtTOR* were grown for 50 DAG under short days with well-watered conditions, and then water was withheld for an additional 14 days. Rosette leaf samples for all three genotypes under both well-watered and drought-challenged conditions were collected at 62 DAGs for RNA isolation. Total RNA for each sample was then extracted using the RNAqueous Total RNA Isolation Kit (Ambion, Catalog# AM1912) following the provided protocol with four biological replicates for each genotype. The extracted RNA was treated with DNase I (Thermo Fisher Scientific), and reverse transcription was performed using the SuperScript IV VILO system (Thermo Fisher Scientific) according to the manufacturer’s instructions. Transcript abundance was measured using the QX200 ddPCR System (Bio-Rad). In brief, each 20 μl of 1x ddPCR SuperMix of probe reaction mixture (no dUTPs, Bio-Rad) containing cDNA templates, forward and reverse primers, and specific probes with optimized concentration was mixed with 70 μl of Droplet Generation oil for Probes in a DG8 Cartridge (Bio-Rad). The cartridge was covered with a DG8 gasket and loaded into the QX200 Droplet Generator (Bio-Rad) to generate PCR droplets. From each droplet mixture, 40 μl was transferred to a 96-well PCR plate and sealed using a PX1 PCR Plate Sealer (Bio-Rad). PCR thermal cycling was optimized, and amplification signals were read using the QX200 Droplet Reader and analyzed using QuantaSoft software (Bio-Rad).

### Western blot analysis

The *GC::AtTOR* and *35S::AtTOR* lines were grown with WT plants for 27 days with adequate water, followed by an 11-day drought condition. After 11 days of drought stress, the whole plant tissue was collected from all the lines for protein extraction. Total protein was extracted from the drought-treated plants and their corresponding unstressed control plants using the phenol extraction method described in (20). The protein was extracted in 2 ml of extraction buffer (1M Tris HCl, 50 mM EDTA, 0.1 M KCl, 0.7 M Sucrose and 0.2% β-Mercaptoethanol, phosphatase inhibitor cocktail-3) and the protein pellet was re-suspended in the rehydration buffer (7 M urea, 2 M thiourea, 4% CHAPS and 100 mM DTT). Protein concentrations were quantified using the Bradford method.

For detection of S6K1 phosphorylation, 20 μg of total protein was used for running SDS-PAGE and blotting using phospho-anti-S6K1-Thr(P)-449 (with 1:5000 dilutions, Catalog# ab207399;, Abcam) and 50 μg was used to detect the TOR expression levels with anti-TOR antibody (1: 5000 dilutions, Catalog# AS12 2608, Agrisera). The Anti-Actin antibody (1:5000 dilutions, Catalog# ab197345, Abcam) was used as a loading control, and HRP-conjugated goat-anti-rabbit secondary IgG antibody (1:10,000 dilutions, Catalog# 205718, Abcam) was used for immunodetection by a chemiluminescent method (ChemiDoc XRS, Bio-Rad). Image J software was used to analyze the relative S6K1 phosphorylation and relative expression of TOR compared with the expression of Actin.

## Data availability

All data described are contained within the document. RNA-seq raw and processed data generated from this study have been deposited into the Gene Expression Omnibus of the National Center for Biotechnology Information (NCBI) under accession GSE292373.

## Supporting information

This article contains supporting information.

## Conflict of interest

The authors declare that they have no conflicts of interest with the contents of this article.
