# Supplementary material for: Guard cell and whole plant expression of AtTOR improves performance under drought and enhances water use efficiency
Source: J Biol Chem. 2025 May 13;301(6):110220. doi: 10.1016/j.jbc.2025.110220 (PMC12181022; doi:10.1016/j.jbc.2025.110220)
Supplement: Table S1 [file mmc1.docx]

**Supplemental Table S1. Primers and probes used in this study.**
