# Supplementary material for: Guard cell and whole plant expression of AtTOR improves performance under drought and enhances water use efficiency
Source: J Biol Chem. 2025 May 13;301(6):110220. doi: 10.1016/j.jbc.2025.110220 (PMC12181022; doi:10.1016/j.jbc.2025.110220)
Supplement: Movie S1 [file mmc2.pptx]

## Slide 1
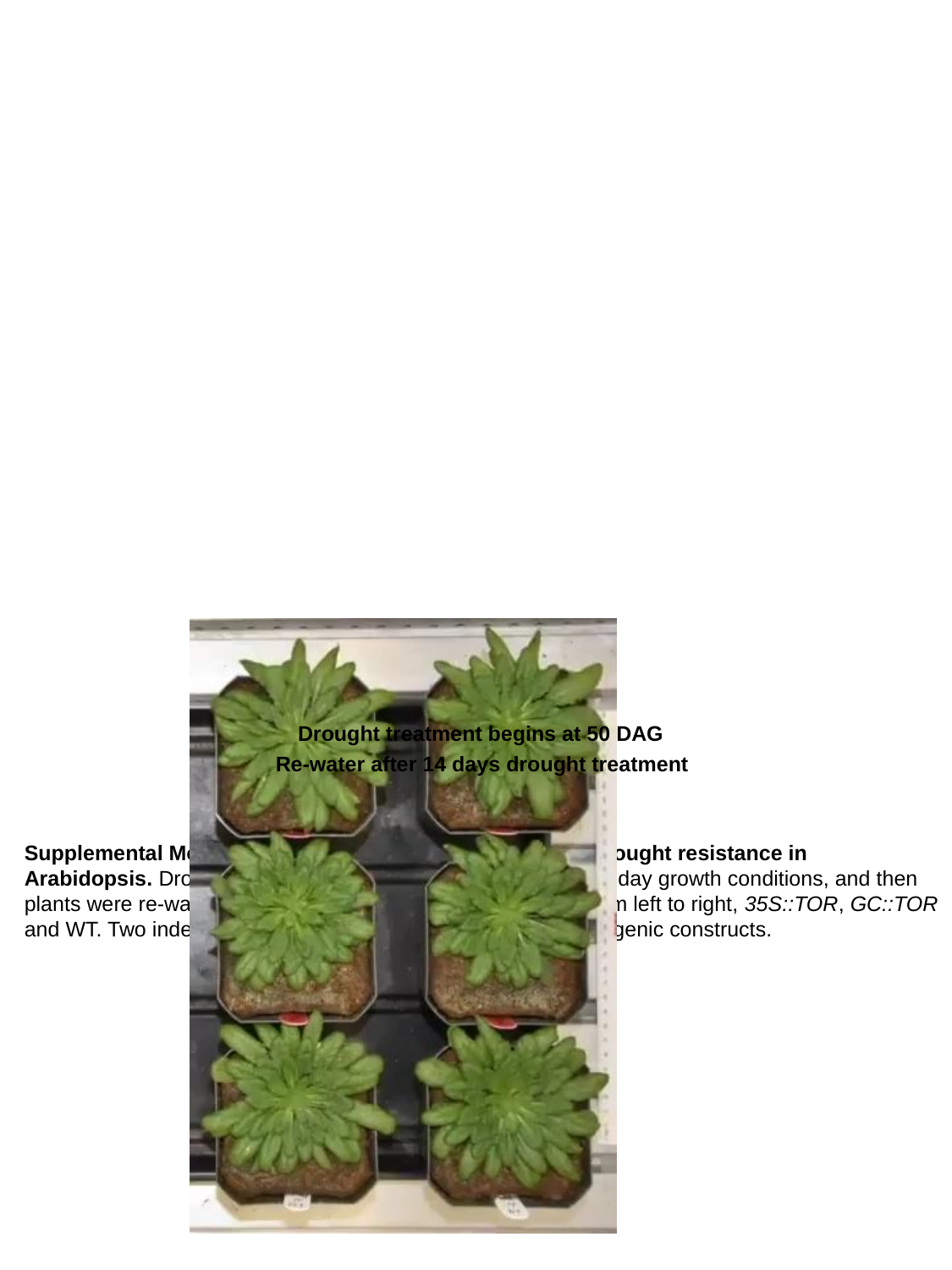

GC::TOR
WT
35S::TOR
Drought treatment begins at 50 DAG
Re-water after 14 days drought treatment
Supplemental Movie S1. TOR transgenic plants improve drought resistance in Arabidopsis. Drought treatment began at 50 DAG under short day growth conditions, and then plants were re-watered after 14 days of drought treatment. From left to right, 35S::TOR, GC::TOR and WT. Two independent lines are presented for the two transgenic constructs.
