# Supplementary material for: Guard cell and whole plant expression of AtTOR improves performance under drought and enhances water use efficiency
Source: J Biol Chem. 2025 May 13;301(6):110220. doi: 10.1016/j.jbc.2025.110220 (PMC12181022; doi:10.1016/j.jbc.2025.110220)
Supplement: Figs. S1-S9 [file mmc3.docx]

**SUPPORTING INFORMATION**


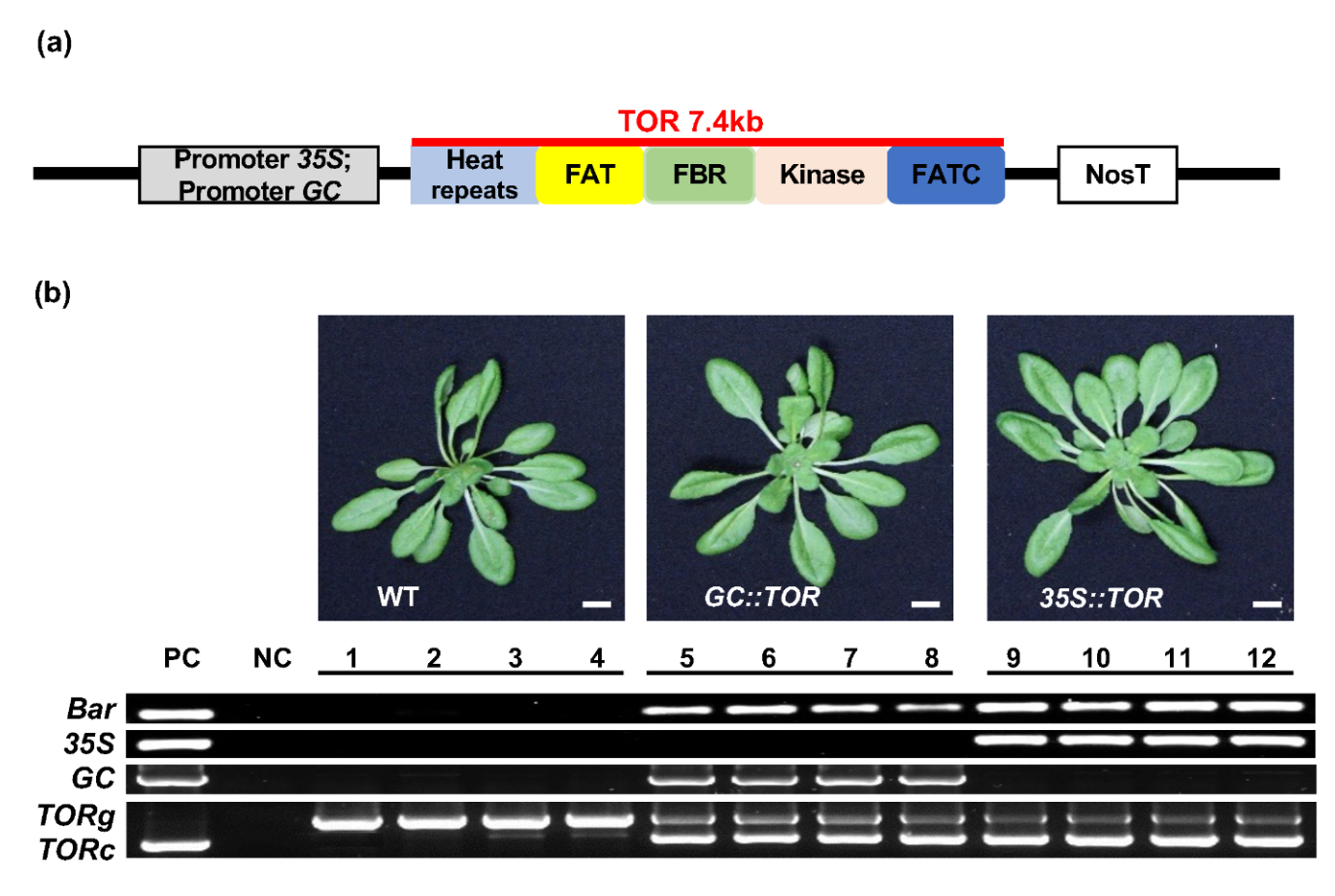


**Supplemental Figure 1. Selection and molecular screening of transgenic plants overexpressing the *AtTOR* gene (7.4kb) driven by either the *35S* promoter or the *Nicotina tabacum* guard cell (*GC*) promoter. (a)** Schematic structure of the vector for transforming transgenic Arabidopsis plants with the *AtTOR* gene. The full-length *AtTOR* cDNA (7.4 kb in size) coding sequence from *Arabidopsis thaliana* was used. For the transgenic Arabidopsis overexpression lines, a guard cell promoter from *Nicotiana tabacum* (*Ntab- TN90*_scaffold3543) or the *35S* promoter were fused to the full-length *AtTOR* gene that had originally been cloned into the p8GWN vector at NotI/XmaI sites and was then transformed into the binary vector, pEarleyGate 203. **(b)** 35 DAG Arabidopsis plants grown under short-day conditions (8 h light) and screened for transformants based on resistance to the herbicide, phosphinothricin (10 mg/L), in the selection medium. The gel depicts PCR products from WT and transgenic plants expressing the *bar* gene (323 bp), *35S* promoter (388 bp), *GC* promoter (210 bp), the *AtTOR* gene (*TOR* cDNA [*TORc*]) which is 534 bp in size) and the *TOR* gene (*TOR* gDNA[*TORg*]) which is 983 bp in size). The columns numbered 1-4 are from WT plants (with a representative WT plant above the numbers), numbers 5-8 are from transgenic plants expressing *GC:AtTOR* (with a representative *GC::AtTOR* plant above the numbers), and numbers 9-12 are from transgenic plants expressing *35S::AtTOR* (with a representative *35S::AtTOR* plant above the numbers). PC and NC labels indicate positive and negative controls, respectively, and *GC* and *35S* denote the *GC* and *35S* promoters. Scale bar for Arabidopsis plant images =1 cm.


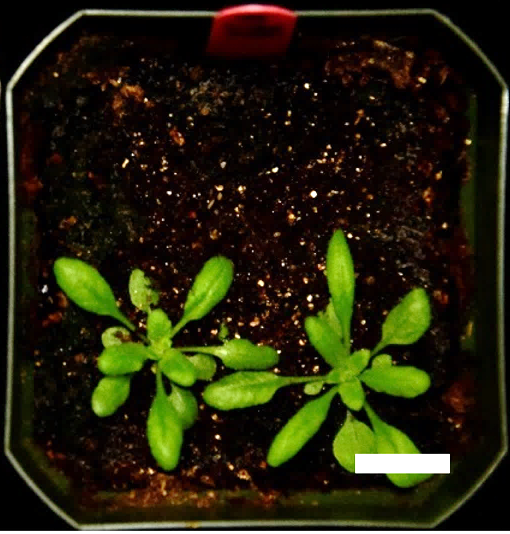

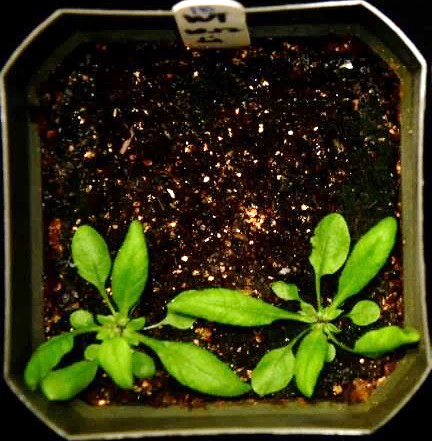

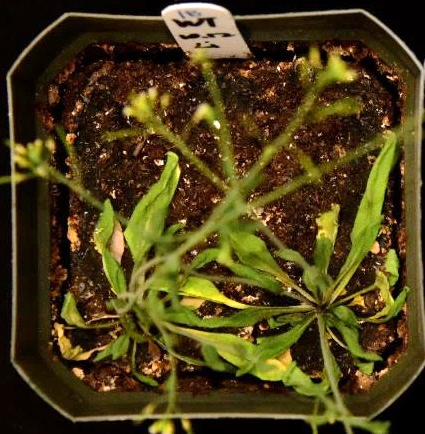

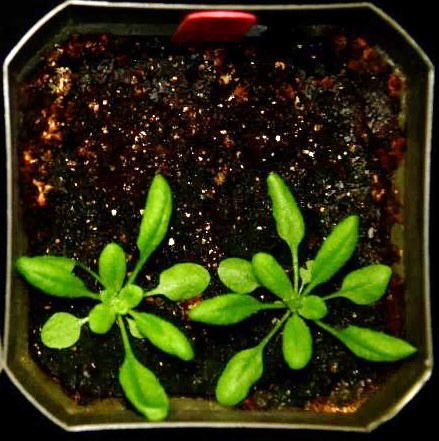

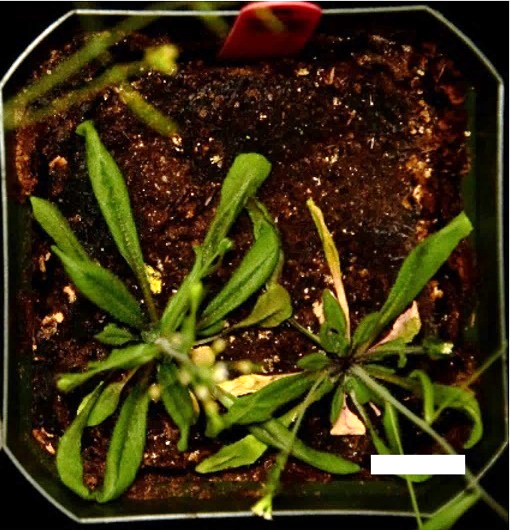

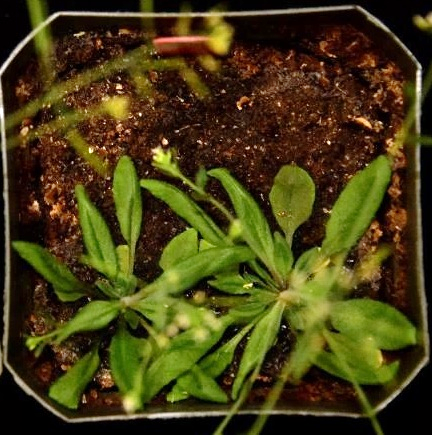


**WT**

***GC::TOR***

***35S::TOR***

**Start drought**

**treatment**

**(27 DAG)**

**Under drought**

**treatment**

**(11 days)**

**Supplemental Figure 2. Screening of full-length *AtTOR* transgenic lines** **expressing either *35S::AtTOR* or *GC::AtTOR* for drought resistance.** WT, *GC::AtTOR* and *35S::AtTOR* plants were grown under well-watered conditions for 27 DAG and then water was withheld for 11 days, under long-day growth conditions (16 h light), and photographed both just before drought stress was initiated (top row), and after drought stress was completed (bottom row). Scale bar = 2.5 mm.


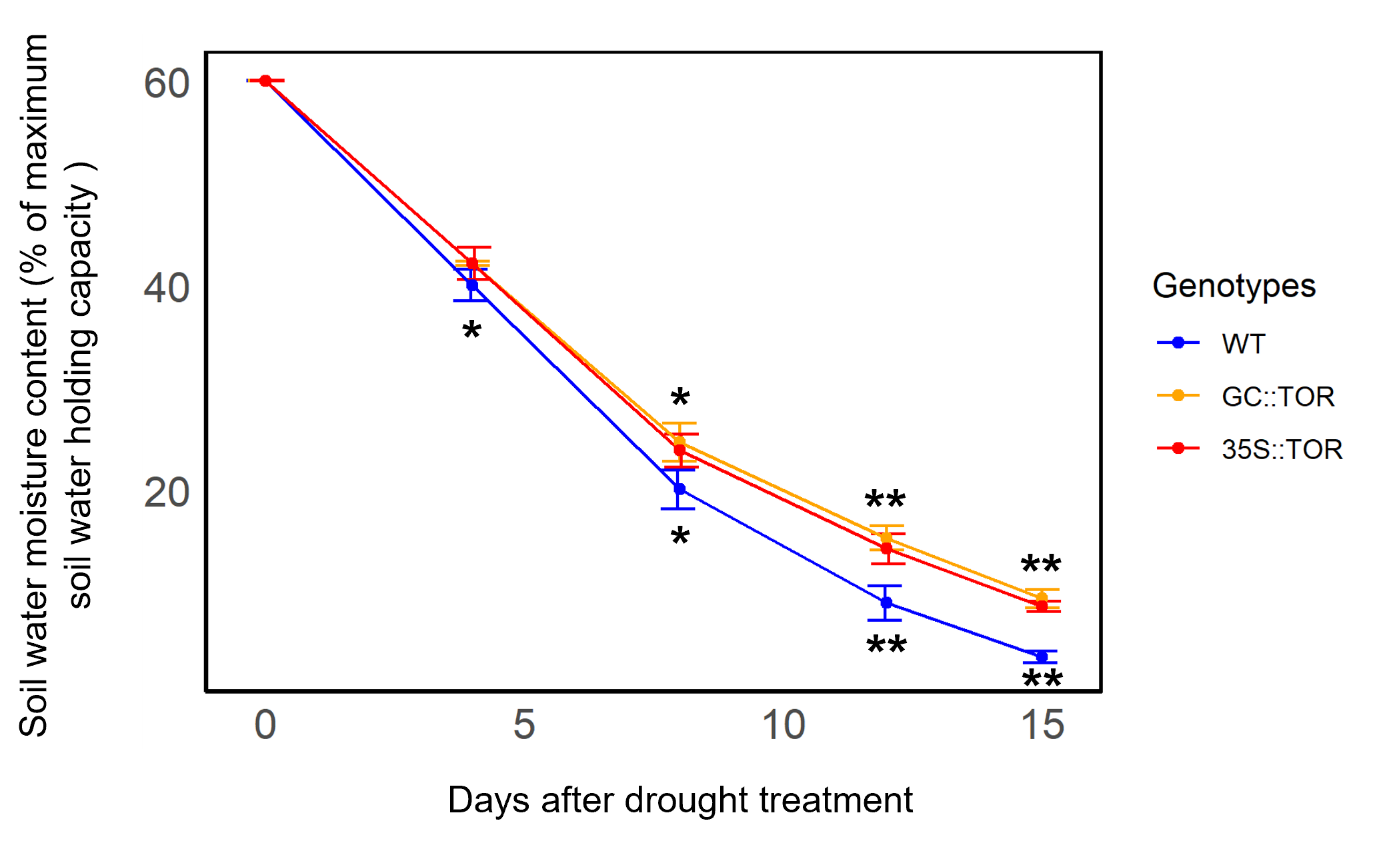


**Supplemental Figure 3. Soil moisture content in pots of WT and *AtTOR* ectopic expression transgenic lines under drought treatment.** Time course for soil water content for intact plants grown in potting mix over 15 days of drought (water withheld starting at day 0 shown on graph). When the drought treatment was started, the soil water content of each pot was set to 60% of full water holding capacity, which was the soil water content for the well-watered plants just before the start of withholding water from the pots for WT, *35S::AtTOR* and *GC::AtTOR* lines. X-axis, days after drought treatment; Y-axis, soil water content as a percentage of 100% water holding capacity. *, 0.01<p<0.05; **, 0.001<p<0.01.


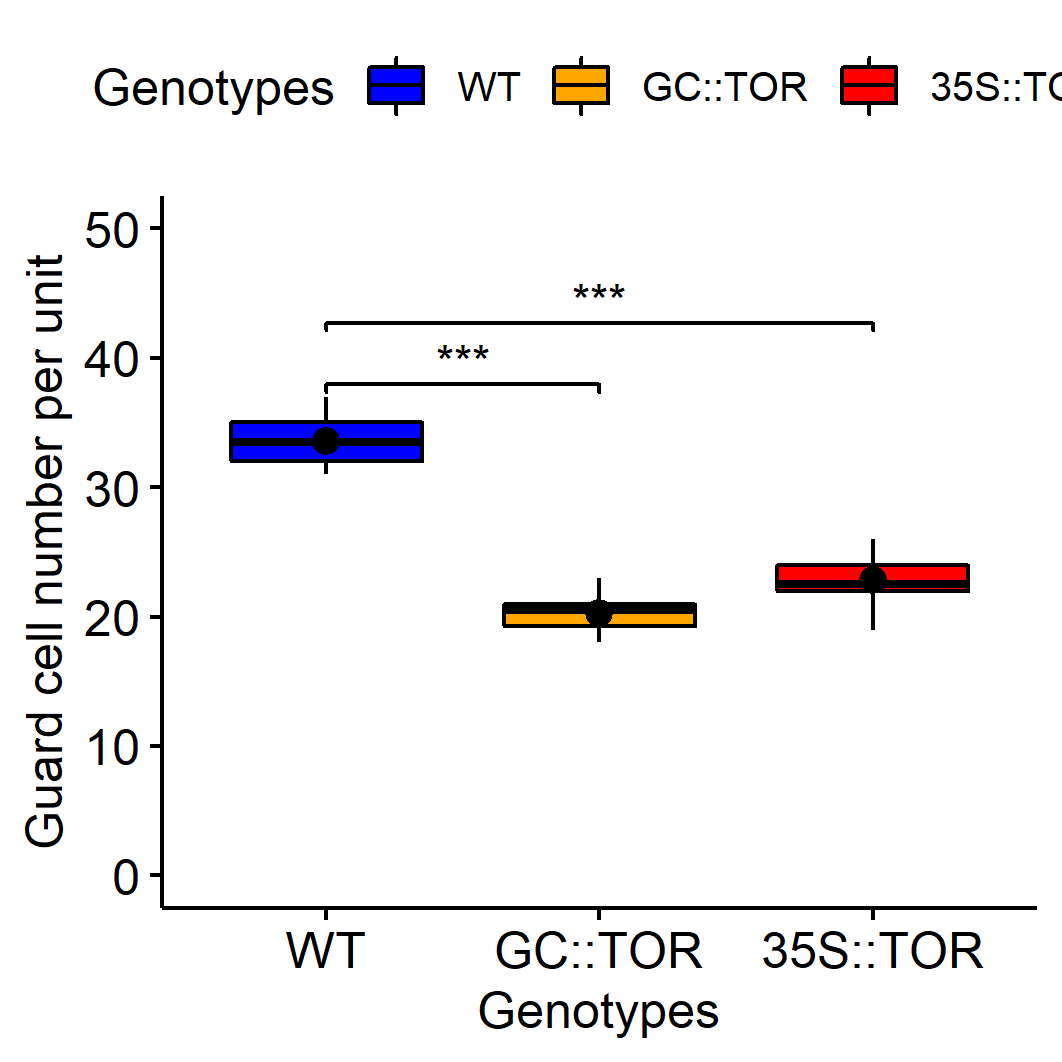


**Supplemental Figure 4. Guard cell density on the abaxial leaf surface of WT and *AtTOR* ectopic expression transgenic lines under well-watered conditions.** Guard cell numbers per a unit of area on the abaxial leaf surface in WT, *35S::AtTOR* and *GC::AtTOR* lines. X-axis, the three genotypes; Y-axis, guard cell numbers per unit of leaf area. One unit represents the standard vision area at 40X magnification using a microscope. ***, p<0.001.


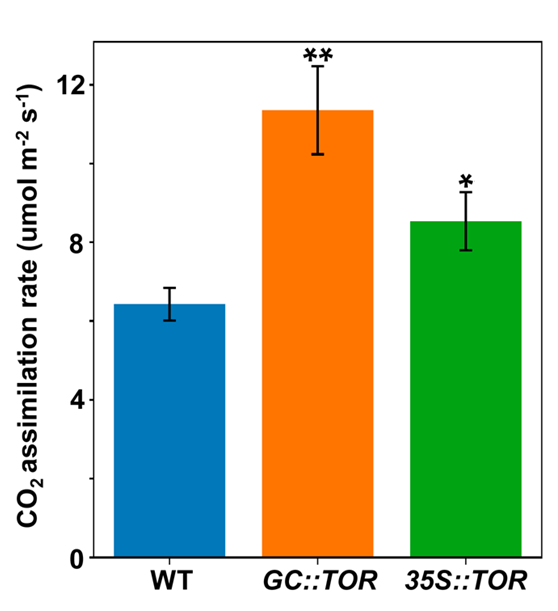


**Supplemental Figure 5. Leaf CO_2_ assimilation for WT and *AtTOR* ectopic expression transgenic lines under well-watered conditions.** Infrared gas exchange analysis of photosynthesis was made via measurement of leaf carbon assimilation using the LI-COR Biosciences 6800 system. The photosynthetic C assimilation rate (GasEX_A) was determined showing the ectopic expression *AtTOR* transgenic lines exhibited higher photosynthetic CO2 assimilation rates than WT plants and the differences are statistically significant (Student’s t-test). **, p<0.01; *, p<0.05.


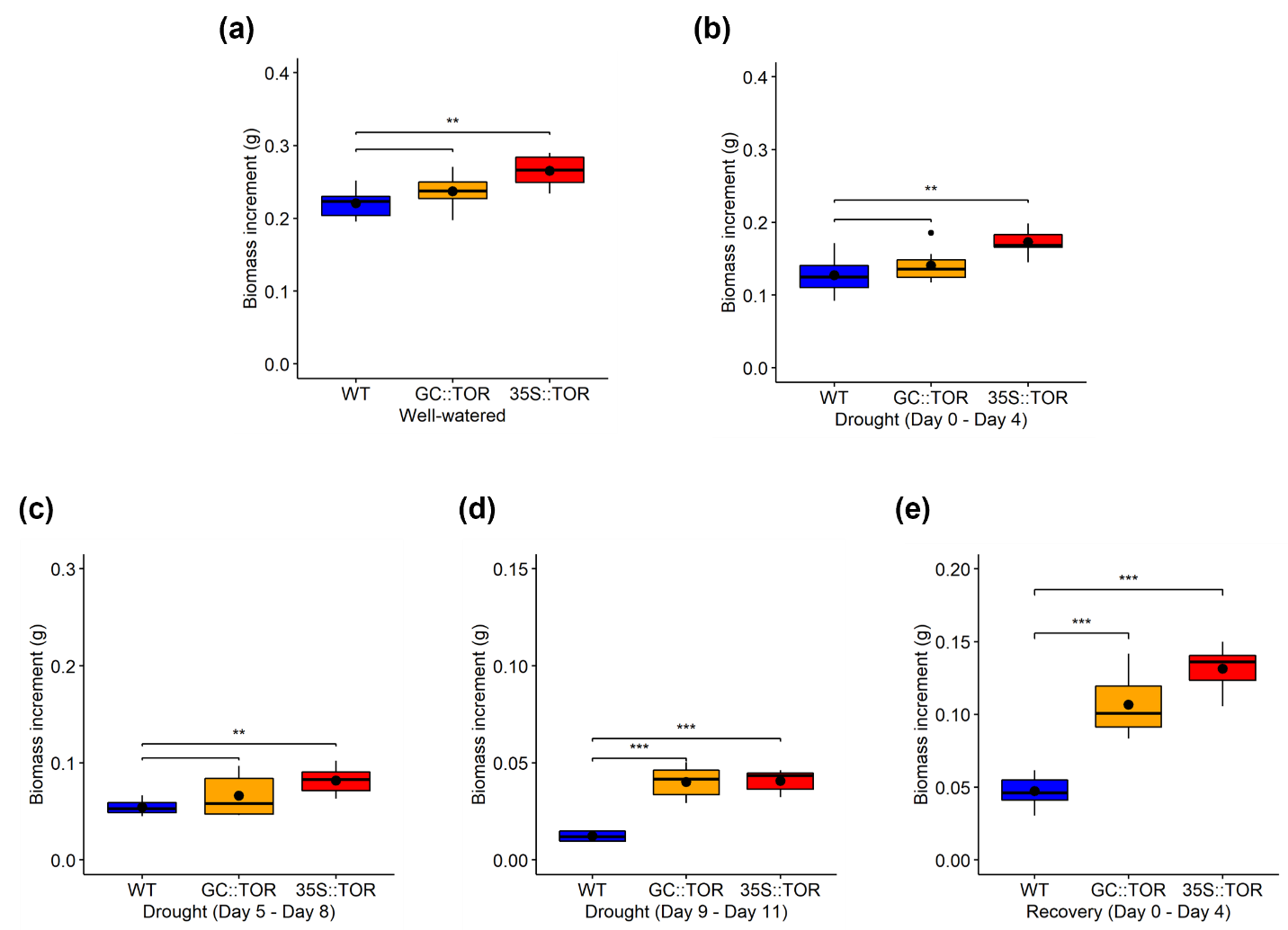


**Supplemental Figure 6. Shoot biomass increase measured for each treatment period (well-watered: 19-27 DAG; drought treatment: days 0-4 of drought [27-31 DAG]; drought treatment: days 5-8 of drought [31-35 DAG]; drought treatment: days 9-11 of drought [35-38 DAG]; and rewatering treatment: days 0–4 [ 39-42 DAG]) in WT, and *AtTOR* ectopic expression transgenic lines. (a)** Boxplots of shoot biomass increase for last 8 days of 27 initial days of well-watered conditions. (19–27 DAG). **(b-d)** Boxplots of shoot biomass increase under drought treatment. Water was withheld on 27DAG, biomass was measured at the end of first 4 days of drought treatment (**b**, 27 DAG-31 DAG), end of Day 5-8 of drought (**c**, 31-35 DAG) and at end of Day 9-11 of drought (**d**, 35-38 DAG), respectively. Plants were rewatered starting on 38DAG. **(e)** Boxplots of shoot biomass measured after 4 days recovery due to rewatering. *, 0.01<p<0.05; **, 0.001<p<0.01; ***, p<0.001.


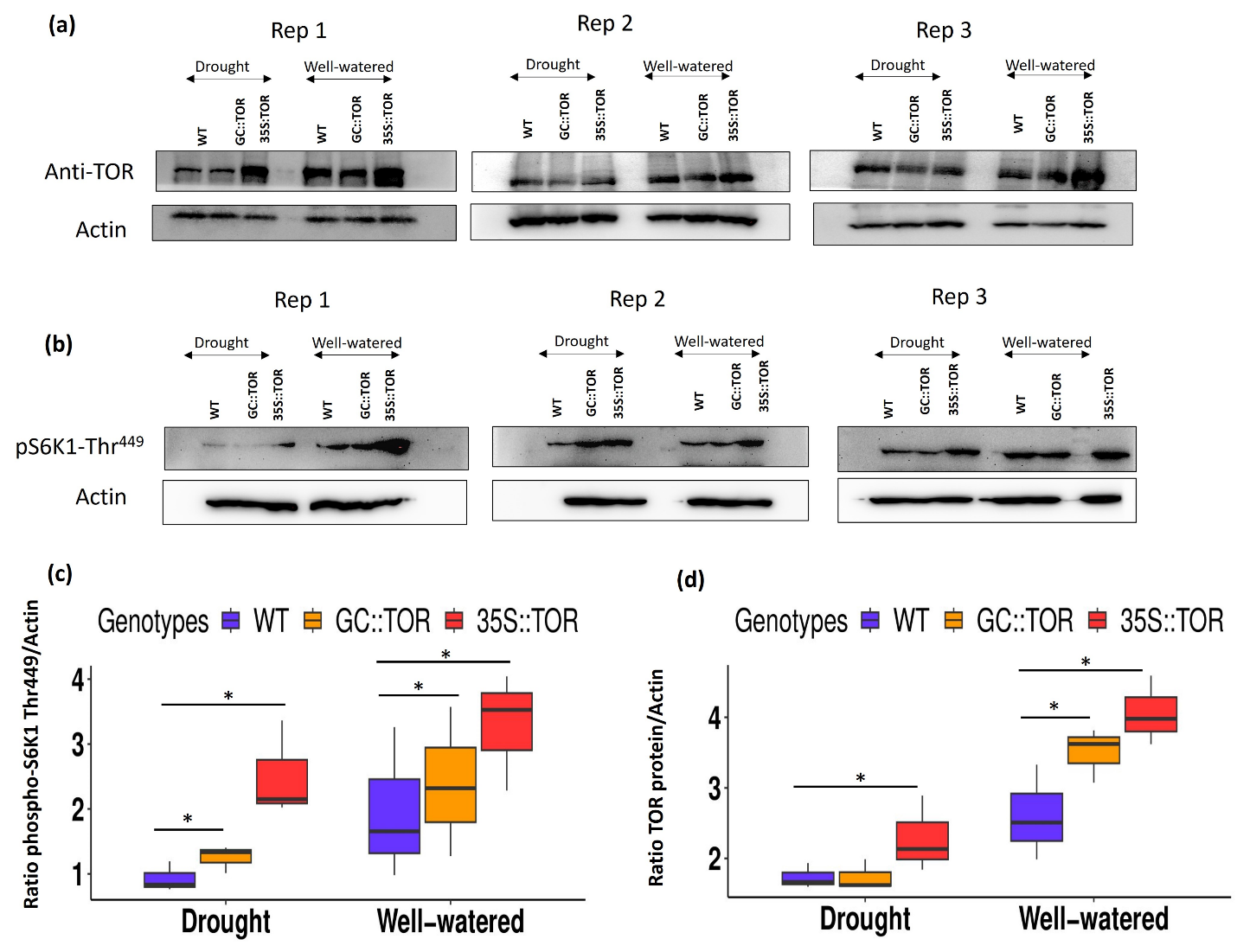
**Supplemental Figure 7. Western blots showing three biological replicates for TOR activity (S6K1 Phosphorylation) and TOR protein expression in WT, *GC*:*AtTOR* and *35S*:*AtTOR* lines after drought treatment.**

(a) Depicts the blots developed from three biological replicates for TOR protein expression level using the anti-TOR antibody (Catalog# AS12 2608, Agrisera) for well-watered and drought-stressed plants. Replicate blot 1 was used as the representative Western blot for TOR protein abundance in Fig. 7b. (b) Three biological replicates for S6K1 phosphorylation detection using phospho-anti-70S6K1-Thr(P)^449^ (Catalog# ab207399, Abcam). Replicate blot 2 was used as the representative TOR kinase activity blot in Fig. 7a. (c, d). Quantification of intensity of signals from S6K1 phosphorylation and TOR expression normalized and represented as the ratio of TOR phosphorylation to Actin protein abundance (Catalog# ab197345, Abcam). The data shown in the graphs (c, d) are represented as the mean of three independent biological replicates ± SE. One way ANOVA was performed and significant values at p<0.05 are represented with asterisks “*”.

**
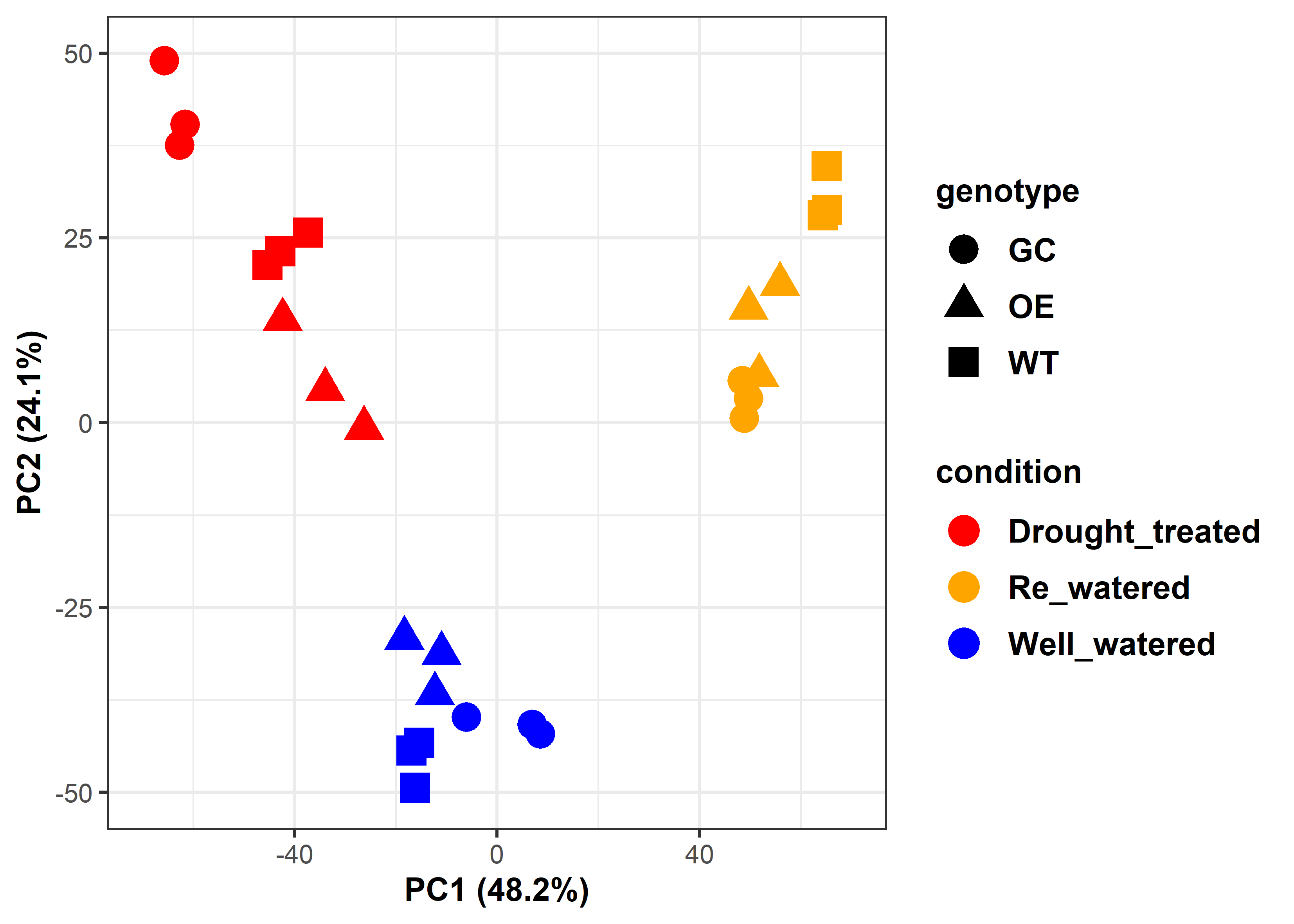
**

**Supplemental Figure 8. Principal component analysis depicting the relationships of the transcriptomes from the three Arabidopsis genotypes in response to watered, drought stressed and recovery from drought stress conditions.** Principal component analysis (PCA), using expressed genes in the combination of 3 genotypes by 3 water treatments. Genotypes are WT, *GC::AtTOR* and *35S::AtTOR* (labeled as OE in the figure key). Treatments are well-watered, drought treated and re-watered recovery from drought. Hence, there are 9 combinations of genotypes x treatments. X-axis, PC1; Y-axis, PC2. The proportion of variance for each principal component is indicated in brackets in the X and Y axis labels. The 3 different genotypes are represented by the 3 symbol shapes in the figure key, and different treatments are represented by the red, yellow and blue colors. Note that the different water treatments are the major factor generating the three separated groups.


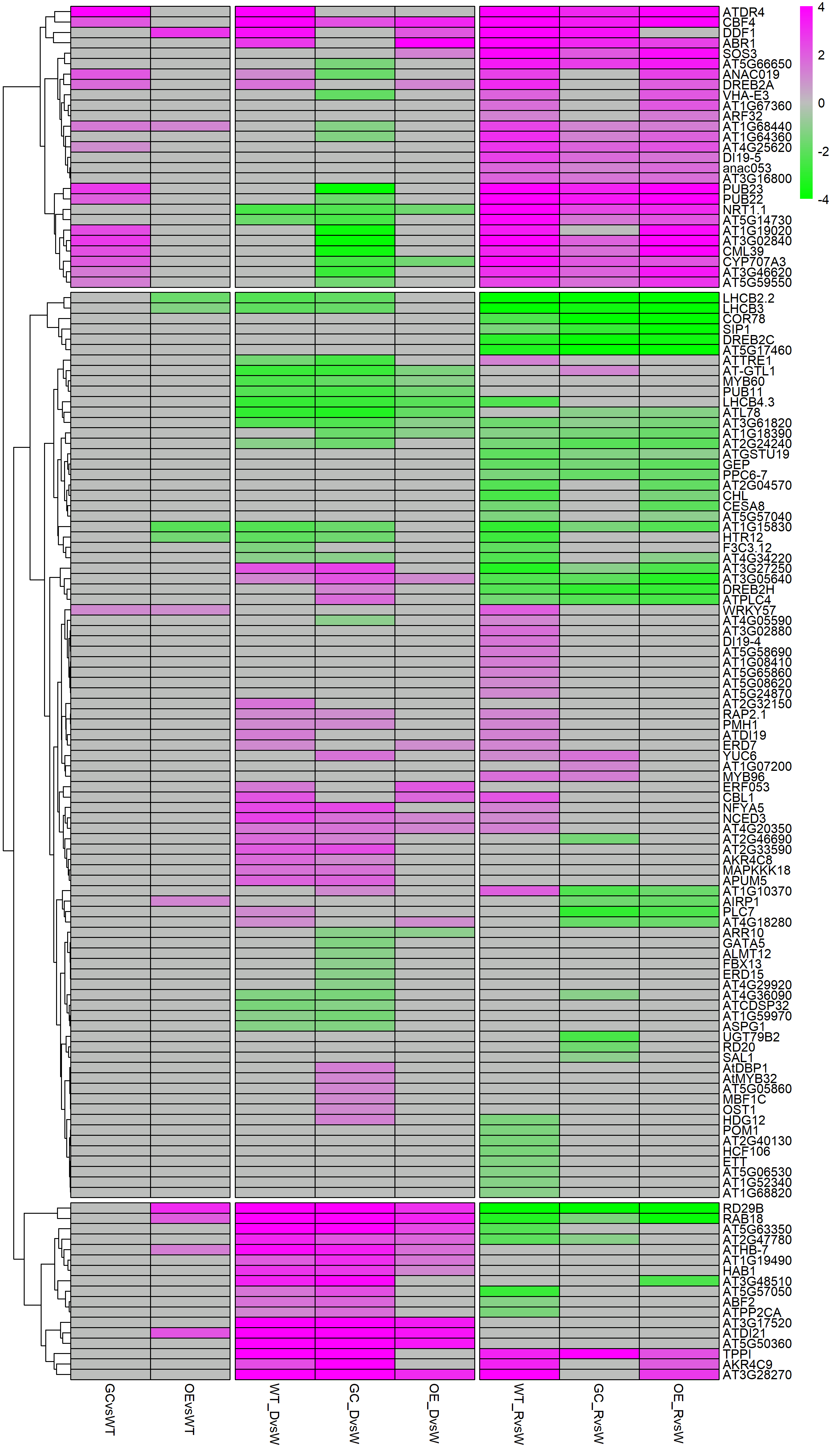


**Supplemental Figure 9. Cluster analysis of all differentially expressed genes (DEGs) induced by well-watered and drought stress, in WT and transgenic plants.** Heatmaps of clustered DEGs with similar expression patterns were generated. The gene clustering is shown by the tree along the left side of the heat map. At least three major clusters were identified, separated by Z-score transformations of expression that was performed for each gene across all samples under different treatments. Expression levels were indicated by the color scheme, from magenta (high expression) to grey (little change in expression), to green (low expression). in response to the indicated treatments, defined below the heat map. WT: wild type; GC: GC::*AtTOR*:*TOR* gene driven by the guard cell-specific promoter; OE: 35S::*AtTOR*: *TOR* gene driven by the 35S promoter; W: Well-watered; D: Drought treatment; R: Re-watered recovery after drought treatment.
